# Wash-Free Bacterial Gram-Typing and Photodynamic Inactivation with Long-Chain-Tailed BODIPY Derivatives

**DOI:** 10.34133/bmr.0069

**Published:** 2024-09-03

**Authors:** Yuefeng Ji, Jigai Li, Chunping Chen, Chunxiang Piao, Xin Zhou, Juyoung Yoon

**Affiliations:** ^1^ College of Agriculture, Yanbian University, Yanji, 133002, China.; ^2^ College of Chemistry and Chemical Engineering, Qingdao University, Qingdao, 266071, China.; ^3^ Department of Chemistry and Nano Science, Ewha Womans University, Seoul, 120-750, Korea.

## Abstract

The rapid identification of bacterial Gram types and their viability, as well as efficient bacterial elimination are crucial for managing bacterial infections yet present important challenges. In this research, we utilized long-chain-tailed BODIPY derivatives to address these hurdles. Our data indicated that these derivatives can distinguish bacteria types and their viability in aqueous solutions through a concise turn-on fluorescent response. Among them, **B-8** stained both live and dead bacteria, and **B-14** offered a wash-free staining. **B-18** demonstrated the highest affinity to selectively fluorescent label viable gram-positive bacteria with a 53.2-fold fluorescent enhancement. Confocal imaging confirmed that **B-18** can serve as an effective membrane-specific probe for facilitating the typing between gram-negative and gram-positive bacteria in a wash-free manner. Additionally, **B-18** displayed selective photodynamic inactivation at 1 μM toward gram-positive bacteria. In vivo studies variformed the ideal photodynamic therapeutic efficacy of **B-18** against methicillin-resistant *Staphylococcus aureus* in mice wound infections.

## Introduction

With the emergence of multidrug-resistant bacteria, bacterial infections have garnered global attention as a significant threat to the public health [1–3]. The development of innovative methods for rapid bacterial pathogen identification and the creation of new antimicrobial agents are of great importance [4–6]. Over the past decades, considerable efforts have been made in this area; however, existing diagnostic methods are often time-consuming, with culture-based techniques requiring extended wait times for results [7–10]. For example, the Gram stain, while faster, suffers from variability in accuracy [11,12]. There is a clear need for a new approach that provides rapid, precise, and efficient bacterial differentiation.

Fluorescence-based technologies have become increasingly popular in bacterial diagnostics due to their increased sensitivity and ease of use [13–16]. These technologies have facilitated the development of imaging techniques capable of distinguishing between bacterial species based on the structural differences between gram-negative and gram-positive bacteria [17–19]. However, many bacterial imaging techniques often need a washing step to eliminate excess fluorophores, thus reducing background noise and improving accuracy [20]. This additional washing procedure can complicate the staining process and hinder real-time analysis.

Moreover, photodynamic therapy (PDT) is gaining attention as a potential alternative to traditional antimicrobial treatments [21,22]. PDT employs light and photosensitizers (PSs) to generate reactive oxygen species (ROS), which selectively target and eliminate bacteria with minimal side effects, a reduced risk of resistance, and a broad spectrum of activity [23–25]. The combination of fluorescent Gram typing and PDT strategies may provide a rapid and efficient tool for the diagnosis and treatment of bacterial infections.

**Fig. 1.**
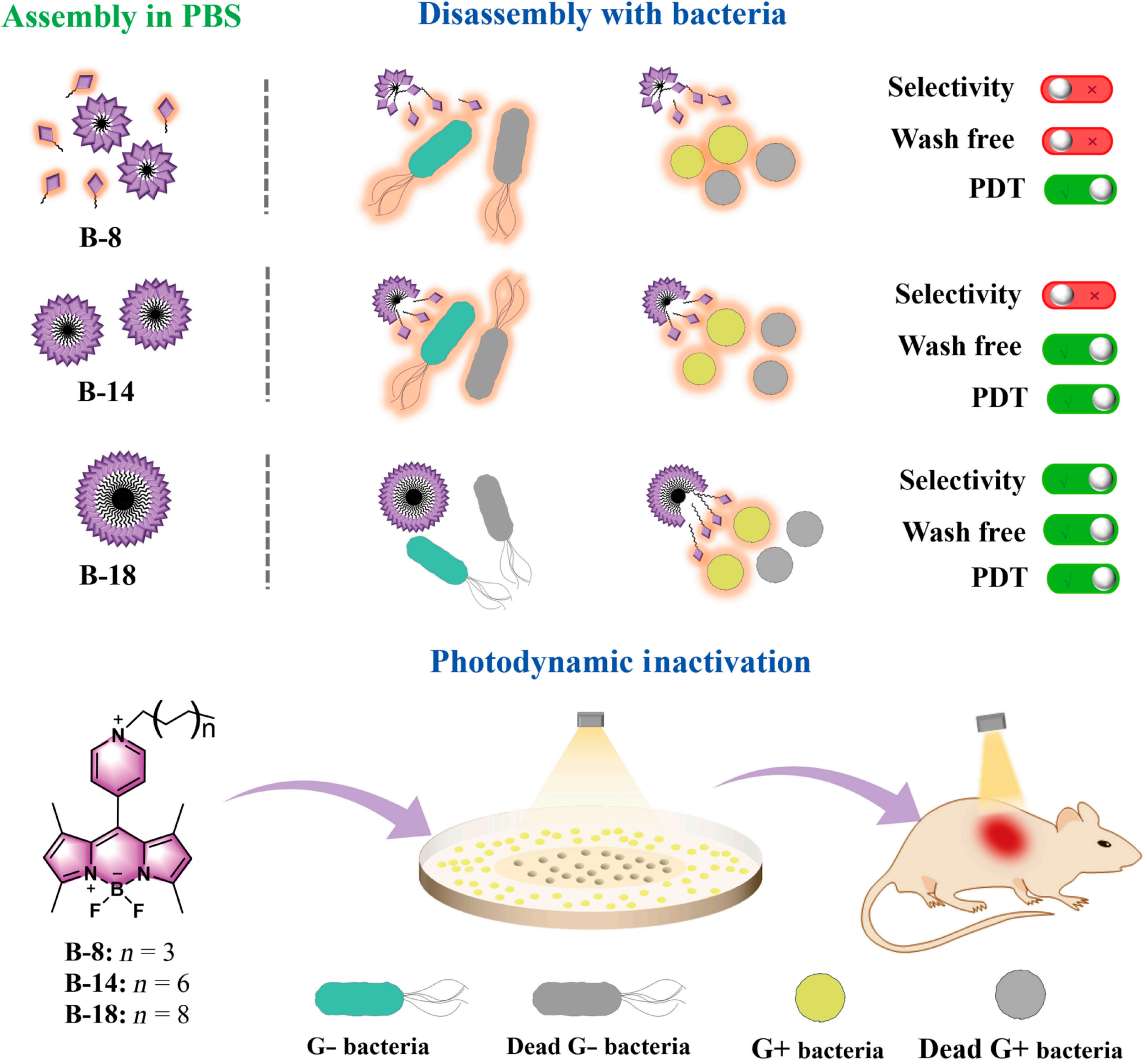
Schematic illustration of selective imaging and photodynamic inactivation of bacteria by compounds (B-8, B-14, and B-18).

Herein, we synthesized and evaluated 3 long-chain-tailed BODIPY derivatives (**B-8**, **B-14**, and **B-18**) for their potential as selective agents for the labeling and photodynamic inactivation of viable gram-positive bacteria (Fig. 1). These compounds feature different self-assembly properties, allowing for selective disassembly upon interaction with bacterial cells, which results in characteristic fluorescence signals. **B-18**, in particular, showed the ability to selectively label viable gram-positive bacteria in a single-step, wash-free process, with a significant increase in fluorescence intensity. Confocal imaging data confirmed the effectiveness of **B-18** as a membrane-specific fluorescent probe for differentiating between gram-negative and gram-positive bacteria. Moreover, both in vitro and in vivo data demonstrated that **B-18** selectively inactivated gram-positive bacteria, with significant therapeutic effects against methicillin-resistant *Staphylococcus aureus* (MRSA) in mice wound infection models.

## Materials and Methods

### Synthesis and photophysical properties

All the solvents and reagents used in this work were of analytical grade. The nutrient agar (NA), Luria-Bertani (LB), and brain-heart infusion medium cultures were purchased from Beijing Land Bridge Technology. *Salmonella enteritidis* (American Type Culture Collection [ATCC] 14028), *Pseudomonas aeruginosa* (BeNa Culture Collection [BNCC] 336458), *Proteus vulgaris Hauser* (BNCC 336633), *Escherichia coli* (ATCC 25922), *Enterococcus faecalis* (BNCC 102668), *Listeria monocytogenes* (BNCC 185986), *Bacillus cereus* (BNCC 1003930), *Staphylococcus aureus* (ATCC 25923), and MRSA (ATCC 29213) were obtained from the Food Research Center of Yanbian University.

^1^H nuclear magnetic resonance (NMR) and ^13^C NMR spectra were recorded on a Bruker Ultrashield 400 MHz and JNM-ECZ600R/S1 600 MHz NMR spectrometer. Mass spectra were recorded using 1200RRLC-6401B instruments. Ultraviolet (UV)/visible (vis) absorption spectra were taken on a Shimadzu UV-2550. Fluorescence (FL) spectra were recorded on Shimadzu RF-6000. Both Zeta potential and dynamic light scattering (DLS) measurements were recorded on Beckman A53878. The morphology of compound aggregates was photographed by a transmission electron microscope (Olympus CKX53). Bacterial morphological structures were recorded in a scanning electron microscope (SEM, Hitachi Regulus8100). Bacterial optical density at 600 nm (OD_600_) was recorded using a multifunctional microplate reader (Tecan Spark20M). Fluorescence imaging was conducted with a confocal laser scanning microscope (CLSM, Nikon C2plus).

### Synthesis

Compound B: 2, 4-dimethylpyrrole (0.34 ml, 3.3 mmol) was added to the stirred solution of 4-pyridinaldehyde (0.14 ml, 1.5 mmol) in 45 ml of CH_2_Cl_2_ (DCM). Then, 50 μl of trifluoroacetic acid was added as the catalyst, and the mixture was stirred for 12 h at room temperature under dark conditions. DDQ (340.5 mg, 1.5 mmol) dissolved in 50 ml of tetrahydrofuran was added to the reaction for 4 h at room temperature. Then, 2 ml of triethylamine was added under the ice bath condition for 10 min. Then, 2 ml of boron trifluoride diethyl etherate was added. The reaction mixture was further stirred for 2 h at room temperature and then extracted with DCM. After the solvent was evaporated under reduced pressure, the crude product was further purified by column chromatography (the eluent was petroleum ether: ethyl acetate = 5:1) to obtain the orange solid powder with a yield of 32.2%. ^1^H NMR (400 MHz, CDCl_3_) δ 8.79 (s, 2H), 7.34 (s, 2H), 6.01 (s, 2H), 2.56 (s, 6H), and 1.40 (s, 6H). ^13^C NMR (101 MHz, CDCl_3_) δ 156.51, 150.54, 143.74, 142.65, 137.60, 130.34, 123.40, 121.83, 29.71, and 14.62. electrospray ionization mass spectrometry (ESI-MS): calculated for 325.1562, found: 326.1655.

Compound **B-8**: Compound B (97.5 mg, 0.3mmol) was dissolved in 20 ml of CH_3_CN, and then 0.1 ml of 1-bromooctane was added. The mixture was further heated to reflux for 24 h. After cooling to room temperature, the solvent was evaporated, and the crude product was purified by flash column chromatography (DCM: MeOH = 10:1). The pure product of red solid powder was obtained with a yield of 55%.^1^H NMR (400 MHz, CDCl_3_) δ 9.78 (d, *J* = 5.7 Hz, 2H), 8.03 (d, *J* = 5.7 Hz, 2H), 6.06 (s, 2H), 5.21 (t, *J* = 6.9 Hz, 2H), 2.57 (s, 6H), 2.10 (s, 2H), 1.43 (s, 6H), 1.26 (dd, *J* = 16.5, 8.6 Hz, 10H), and 0.86 (t, *J* = 6.8 Hz, 3H).^13^C NMR (151 MHz, CDCl_3_) δ 158.55, 153.08, 146.39, 142.25, 141.84, 132.74, 129.37, 128.64, 122.98, 62.49, 32.05, 31.66, 29.76, 29.09, 29.02, 25.98, 22.62, 15.57, 14.85, 14.18, and 14.11. ESI-MS: calculated for 438.2887, found: 438.2900.

Compound **B-14**: **B-14** was synthesized according to the same procedure as **B-8**. Yield: 40%. ^1^H NMR (400 MHz, CDCl_3_) ^1^H NMR (400 MHz, CDCl_3_) δ 9.82 (s, 2H), 8.04 (s, 2H), 6.05 (s, 2H), 5.19 (s, 2H), 2.56 (s, 6H), 2.09 (s, 2H), 1.42 (s, 6H), 1.23 (d, *J* = 12.3 Hz, 20H), and 0.87 (s, 3H). ^13^C NMR (151 MHz, CDCl_3_) δ 158.51, 152.99, 146.47, 141.83, 132.80, 129.36, 128.66, 122.95, 62.39, 32.07, 31.98, 29.74, 29.71, 29.66, 29.54, 29.48, 29.41, 29.10, 25.99, 22.74, 15.53, 14.83, and 14.18. ESI-MS: calculated for 522.3826; found: 522.3830.

Compound **B-18**: **B-18** was synthesized according to the same procedure as **B-8**. Yield: 33%. ^1^H NMR (400 MHz, CDCl_3_) δ 9.57 (d, *J* = 6.3 Hz, 2H), 8.04 (d, *J* = 6.2 Hz, 2H), 6.05 (s, 2H), 5.11 (t, *J* = 7.1 Hz, 2H), 2.56 (s, 6H), 2.09 (s, 2H), 1.46 (s, 6H), 1.29 to 1.25 (m,30H), and 0.87 (t, *J* = 6.7 Hz, 3H). ^13^C NMR (151 MHz, CDCl_3_) δ 158.65, 153.31, 145.93, 141.90, 132.49, 129.34, 128.60, 123.01, 77.31, 77.10, 76.89, 62.95), 32.00, 31.74, 29.92 to 29.61, 29.61 to 29.30, 29.06, 25.99, 22.76, 16.02, 14.87, and 14.19. ESI-MS: calculated for 578.4452, found: 578.4462.

### Photophysical properties

The BODIPY derivatives **B-8**, **B-14**, and **B-18** were initially dissolved in ethanol (EtOH) to create a 1 mM stock solution for subsequent analysis. The UV/vis absorption and fluorescence spectra for these compounds, at a final concentration of 10 μM, were then evaluated in a mixture of phosphate-buffered saline (PBS, 10 mM, pH 7.4) and EtOH.

### DLS

DLS was employed to determine the size distribution of the compounds in aqueous and PBS solutions. Concurrently, the zeta potential of compound aggregates in aqueous solution was measured using a Beckman A53878 instrument.

### Bacterial cultivation

*E. coli* and *S. aureus* colonies from NA medium were transferred to 10 ml of LB medium and incubated at 37 °C with 120 rpm shaking for 16 h. Bacterial cells were collected by centrifugation (8,000 rpm, 5 min), washed twice with PBS (pH 7.4), and resuspended to an OD_600_ of 0.5 for further analysis.

### Selectivity of live and dead bacteria

The aforementioned culture procedure was followed for *E. coli* and *S. aureus* (OD_600_ = 0.5). Cells were centrifuged (8,000 rpm, 5 min), resuspended in 75% EtOH with shaking (120 rpm, 1 h, room temperature), and centrifuged again to isolate dead bacteria. These were then resuspended in PBS. Live and dead bacterial samples were incubated with **B-8**, **B-14**, and **B-18** (10 μM each) for 20 min at room temperature, followed by fluorescence intensity measurement upon 508-nm excitation. **B-18**’s interaction with bacteria over time was profiled to assess fluorescence response.

### Fluorescence imaging of live and dead bacteria

Cultures of live and dead bacteria were prepared as described. The concentration was standardized to 1 × 10^6^ CFU/ml. Live and dead samples were then incubated with **B-8**, **B-14**, and **B-18** (10 μM) for 20 min at room temperature. For the washout, samples were centrifuged (2,000 rpm, 5 min) and resuspended in PBS. The washout was omitted for **B-14** and **B-18**. Sytox Deep Red was added for 10 min of dark incubation before imaging. Samples were mounted on confocal imaging dishes with agarose and examined using CLSM.

### Zeta potential measurement

For zeta potential (ζ) assessment, compounds (10 μM) were mixed with bacterial suspension (OD_600_ = 0.5) for 20 min. The mixture was centrifuged (5,000 rpm, 5 min) and washed with deionized water, and ζ-potential was measured using a Beckman A53878 instrument.

### Gram-typing fluorescence imaging

Eight bacterial strains were cultured, including 4 gram-negative (*S. enteritidis*, *P. aeruginosa*, *P. vulgaris*, and *E. coli*) and 4 gram-positive (*E. faecalis*, *L. monocytogenes*, *B. cereus*, and *S. aureus*). **B-8** was incubated with bacteria for 20 min, followed by centrifugation and resuspension in PBS. For **B-14** and **B-18**, the washout was excluded. Samples were prepared on confocal imaging dishes and examined with CLSM.

### Fluorescence imaging of *S. aureus* and *E. coli* with B-18

Bacterial suspensions (OD_600_ = 0.5) were mixed with **B-18** (10 μM) and incubated for 20 min. Prepared slides were examined for fluorescence.

### Detection of *S. aureus* concentrations with B-18

*S. aureus* suspensions that ranged from 1 × 10^9^ to 0 CFU/ml were incubated with **B-18** (10 μM) for 20 min and visualized under UV light, establishing a linear correlation between fluorescence intensity and bacterial concentration.

### Real-time bacterial viability tracking with B-18

*S. aureus*, postresuspension and vancomycin (5 μM) treatment, was incubated for 0 to 4 h. Aliquots were incubated with **B-18** (10 μM) for 20 min, and samples were prepared for CLSM observation.

### General ROS detection

The ROS indicator, 2’,7’-dichlorodihydrofluorescein (**DCFH**), was applied to assess ROS generation by **B-8**, **B-14**, and **B-18** in PBS under white light (20 mW·cm^-2^). **DCFH** fluorescence, induced by ROS sensitized by the compounds (10 μM each), was excited at 485 nm with emissions recorded between 500 and 700 nm. Maximal fluorescence intensity indicated ROS production.

### MIC determination in the dark

The minimum inhibitory concentration (MIC) of **B-8**, **B-14**, and **B-18** was established as the lowest concentration significantly inhibiting bacterial growth. Using the microbroth dilution method, the antibacterial activity was evaluated against *S. aureus* and *E. coli*, with initial compound concentrations at 80 μM in 20-μl volumes. Following a 2-fold serial dilution in a 96-well plate, bacteria were cultured in LB for 24 h and then diluted to approximately 1 × 10^6^ CFU/ml. The diluted bacteria (180 μl) were mixed with compounds, and the plate was incubated at 37 °C, 120 rpm for 24 h. OD_600_ was measured, and experiments were triplicated.

### MIC under white light

The MIC of **B-8**, **B-14**, and **B-18** under white light was determined postdark incubation. Compounds were prepared at 16 μM, and after 30 min of white light exposure (20 mW·cm^−2^), samples were incubated in the dark at 37 °C, 120 rpm for 24 h. OD_600_ measurements were taken, with triplicate experiments.

### In vitro photodynamic bacterial inactivation

Bacterial suspensions (1 × 10^6^ CFU/ml in PBS) were treated with **B-8**, **B-14**, and **B-18** (1 μM for *S. aureus*; 2 μM for **B-8**, 8 μM for **B-14**, and 10 μM for *E. coli*) for 30 min. Mixtures were then spread on NA medium and exposed to white light (20 mW·cm^-2^) for 30 min, followed by incubation at 37 °C for 24 h.

### SEM analysis

After incubation with **B-8**, **B-14**, and **B-18** and light exposure (20 mW·cm^-2^ for 30 min), bacteria were centrifuged (8,000 rpm, 5 min), and the pellet was washed 3 times with PBS. Fixed with 2.5% glutaraldehyde for 8 h, samples were dehydrated through a graded EtOH series and freeze-dried. Samples were then examined using SEM.

### In vivo anti-infection assay

For in vivo experiments, male BALB/c mice aged 8 to 10 weeks with an average weight of 20 g were utilized. All procedures involving animals were approved by the Ethics and Welfare Committee for Animal Experimentation at Yanbian University (1016). An animal model of MRSA infection on the back skin of BALB/c mice was established. The mice were randomly divided into 4 groups: (a) treated with PBS only (control group), (b) treated with **B-8** @white-light, (c) treated with **B-14** @white-light, and (d) treated with **B-18** @white-light. The mice were anesthetized by intraperitoneal injection of 1% pentobarbital sodium physiological saline solution (5 ml/kg). A circular skin area with a diameter of 0.8 cm was removed from the back of each mouse to create a full-thickness skin wound model. The bacterial suspension (100 μl, 1 × 10^8^ CFU/ml) was applied to the wound and left for 30 min. The 5 μM compound solution was sprayed onto the bacteria-infected wound area in PBS for 30 min, followed by a 10-min exposure to white light (20 mW·cm^-2^). The mice were housed separately in different cages, and wound size was recorded using a camera at specified time intervals (0, 3, 7, and 10 d).

### Histological analyses

At day 10 postoperation, tissue histological analysis was conducted on the wound sites. Wound tissues were collected, pinned onto aluminum foil, and fixed in a 4% paraformaldehyde solution for 24 h. Hematoxylin-eosin (HE) and Masson staining were employed for the pathological section analysis of the wound tissues.

### Statistical analysis

The reported values were expressed as mean ± SD with *n* = 3, unless specifically stated. The Origin 2021 software was used for graphic drawing. The statistical significance of differences between groups was performed by using pair-sample *t* testing method on the Origin 2021 software.

## Results

### Synthesis and photophysical properties

**Fig. 2.**
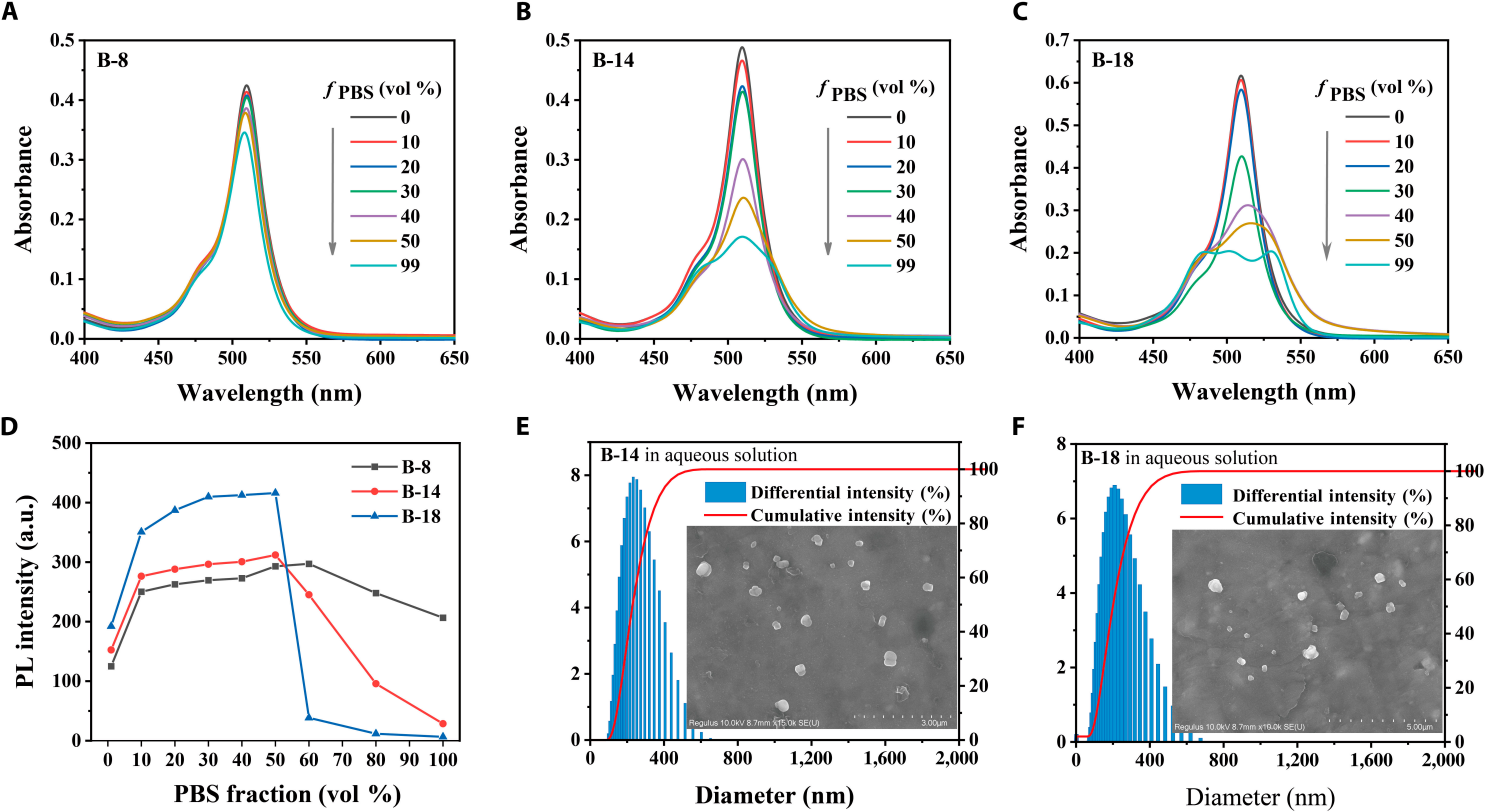
The photophysical properties of BODIPY derivatives. (A to C) UV absorption spectra of B-8, B-14, and B-18 in EtOH/PBS mixtures with different PBS fractions. (D) The plot of relative PL intensity versus *f*_PBS_ in the EtOH/PBS. (E and F) DLS results and SEM image (inset) of the aggregates of B-14 and B-18 in aqueous solution. (concentration: 10 μM).

In this work, we designed and synthesized 3 BODIPY derivatives with long-chain tails (**B-8**, **B-14**, and **B-18**). All compounds are characterized unanimously by ^1^H/^13^C NMR and HRMS (Supplementary Materials, Figs. S1 to S10). The photophysical properties of **B-8**, **B-14**, and **B-18** were firstly investigated in ethanol and PBS mixtures using UV/vis absorption and fluorescence spectroscopy. As shown in Fig. 2A to C, the absorbance of **B-8** showed slightly change with the increasing of PBS fraction (*f*_PBS_) from 0 to 99% (V/V). In contrast, a significant decrease in absorption was observed for **B-14** and **B-18** at a *f*_PBS_ over 60%, with multiple broad peaks present at *f*_PBS_ = 99%. This data indicated the formation of aggregation of **B-14** and **B-18** in aqueous environments. Correspondingly, a sharp decline in fluorescence intensity was observed at *f*_PBS_ = 60% for these compounds, consistent with UV absorption data (Fig. 2D, Fig. S11, and Table S1). Notably, **B-14** and **B-18** displayed no fluorescence at *f*_PBS_ = 99%, suggesting an aggregation-caused quenching (ACQ) effect, characteristic of such long-chain compounds in PBS.

SEM data revealed the presence of spherical aggregates with an average diameter of approximately 200 nm for **B-14** and **B-18** when suspended in aqueous solution (Fig. 2E and F). In contrast, **B-8** was well soluble in aqueous solution. The average sizes of the aggregates for **B-8**, **B-14**, and **B-18** were determined to be 232.2, 271.6, and 476 nm, respectively, which were in agreement with transmission electron microscope data (Fig. S12). A comparison with the SEM data indicated an increase in aggregate size and a broader particle size distribution for **B-8**, **B-14**, and **B-18** in PBS. The difference is likely due to the length variation of hydrophobic alkane chains in the compounds and the impact of salt content on ionic strength, which can neutralize the surface charge of the aggregates, diminishing it and consequently facilitating additional aggregation [26]. As presented in Fig. S13, the zeta potentials of **B-8**, **B-14**, and **B-18** were measured at +6.08, +10.68, and +48.48 mV, respectively, indicating structural stability of the **B-18** aggregates in aqueous media. The positive charge on these aggregates suggests a potential for enhanced interaction with negative charged bacterial.

### Selective identification and imaging of bacteria

**Fig. 3.**
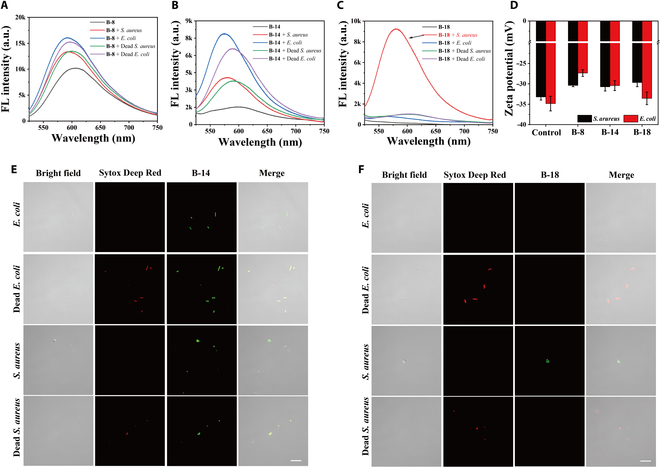
Selective identification and imaging of bacteria by BODIPY derivatives. (A to C) FL spectra of compounds B-8, B-14, and B-18 incubated with live/dead *S. aureus* and *E. coli* with OD_600_ = 0.5 for 20 min (concentration 10 μM, slit width: 5/5 nm, Ex = 508 nm). (D) Zeta potential change after incubation of compound B-8, B-14, and B-18 with *S. aureus* and *E. coli* (concentration 10 μM, OD_600_ = 0.5). (E and F) CLSM imaging of live/dead *S. aureus* and *E. coli* costained with commercially available nucleic acid dye Sytox Deep Red and B-14 and B-18 (scale: 5 μm, wash-free, Ex of B-8, B-14, and B-18: 515 nm, Em: 530 to 560 nm. Ex of Sytox Deep Red: 635 nm, Em: 650 to 750 nm).

**Fig. 4.**
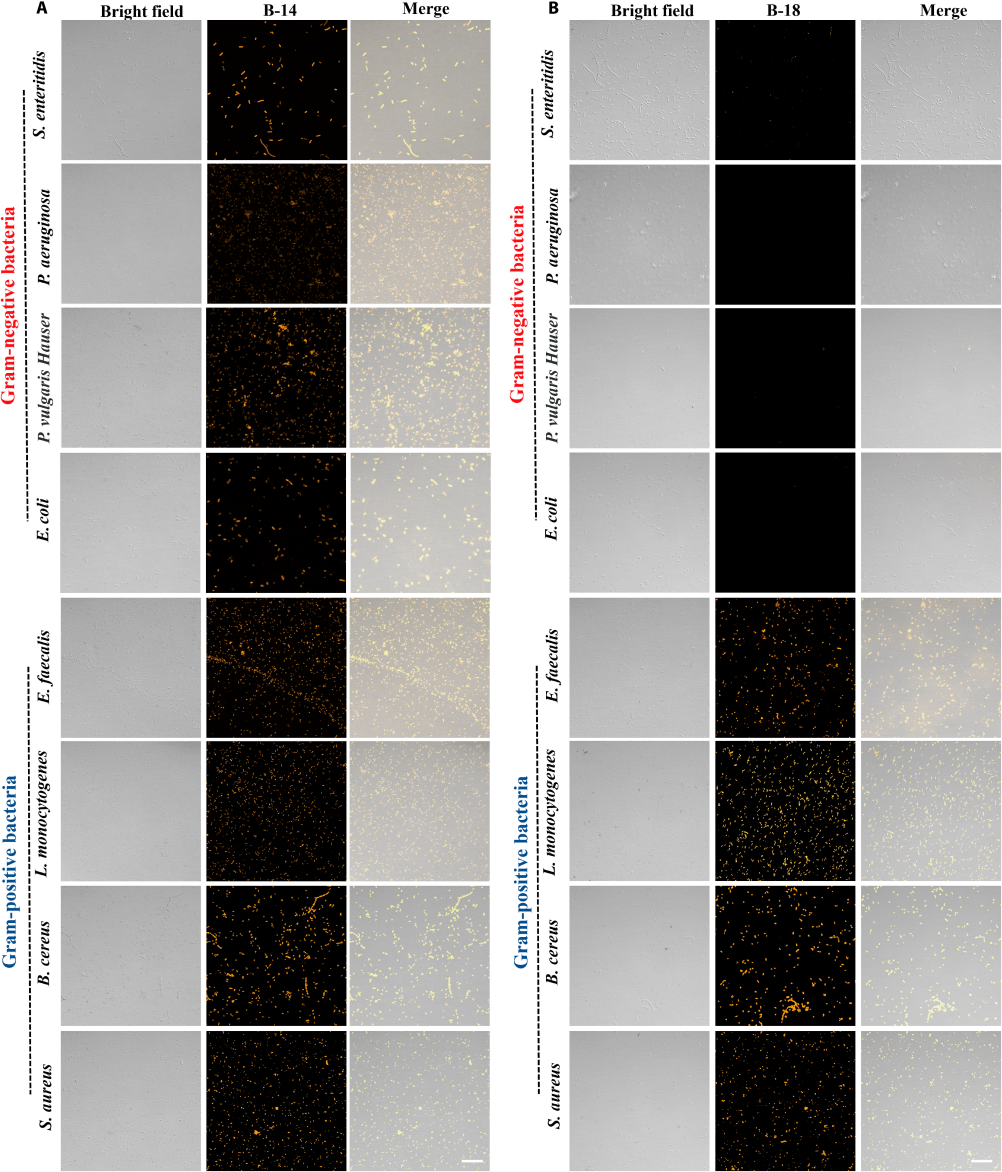
Bacterial fluorescence imaging with BODIPY derivatives. (A and B) CLSM imaging of B-14 and B-18 with gram-negative bacteria (*S. enteritidis*, *P. aeruginosa*, *P. vulgaris*, and *E. coli*) and gram-positive bacteria (*E. faecalis*, *L. monocytogenes*, *B. cereus*, and *S. aureus*) (concentration: 10 μM, scale bar: 10 μm, wash-free, Ex: 515 nm, Em: 550 to 650 nm).

**Fig. 5.**
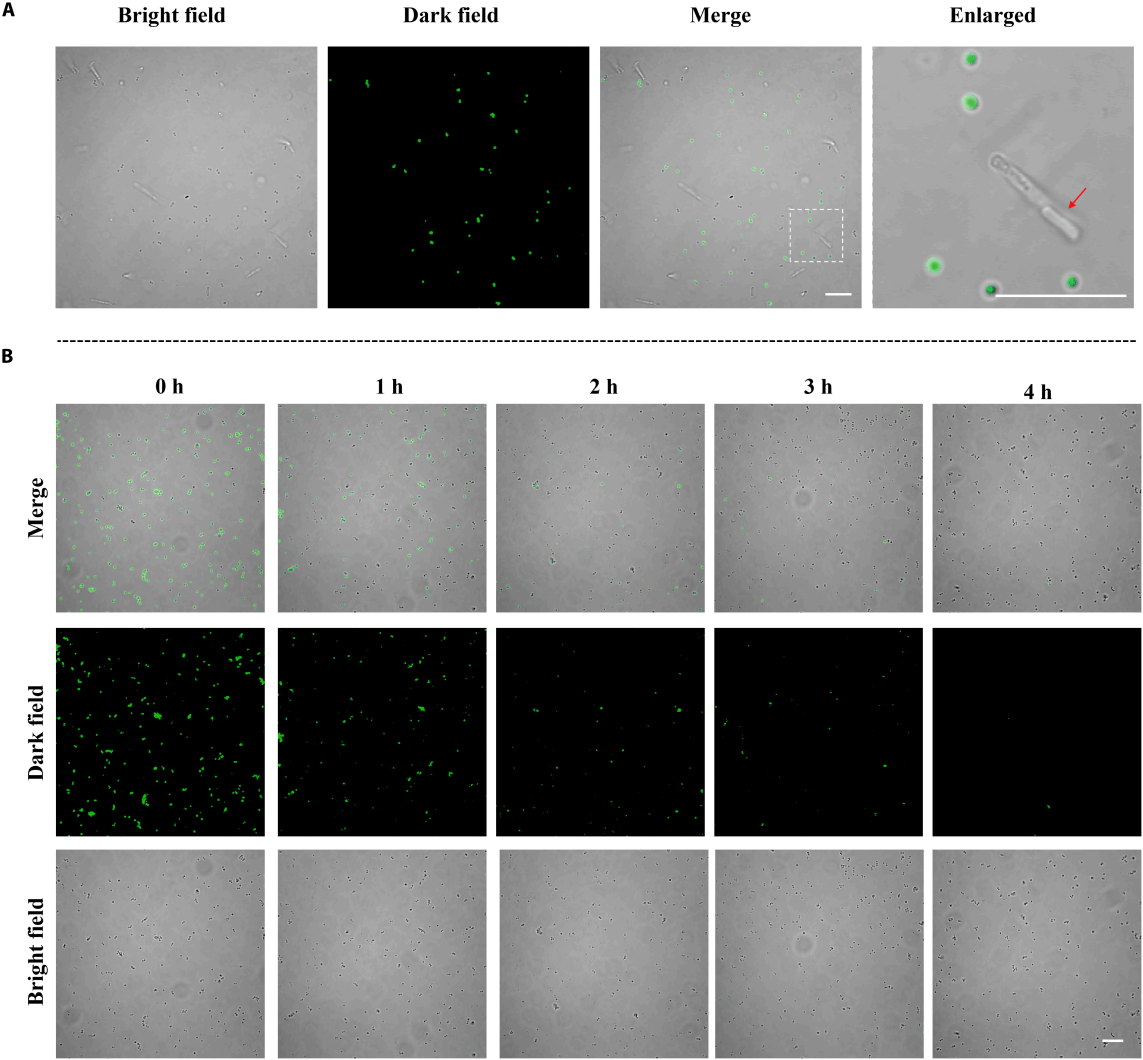
B-18’s gram typing and bacterial viability assessment capabilities. (A) CLSM imaging of *S. aureus* and *E. coli* costained with B-18. (B) CLSM imaging of vancomycin-incubated *S. aureus* costained with B-18 (concentration: 10 μM, scale: 5 μm, wash-free, Ex: 515 nm, Em: 530 to 560 nm).

**Fig. 6.**
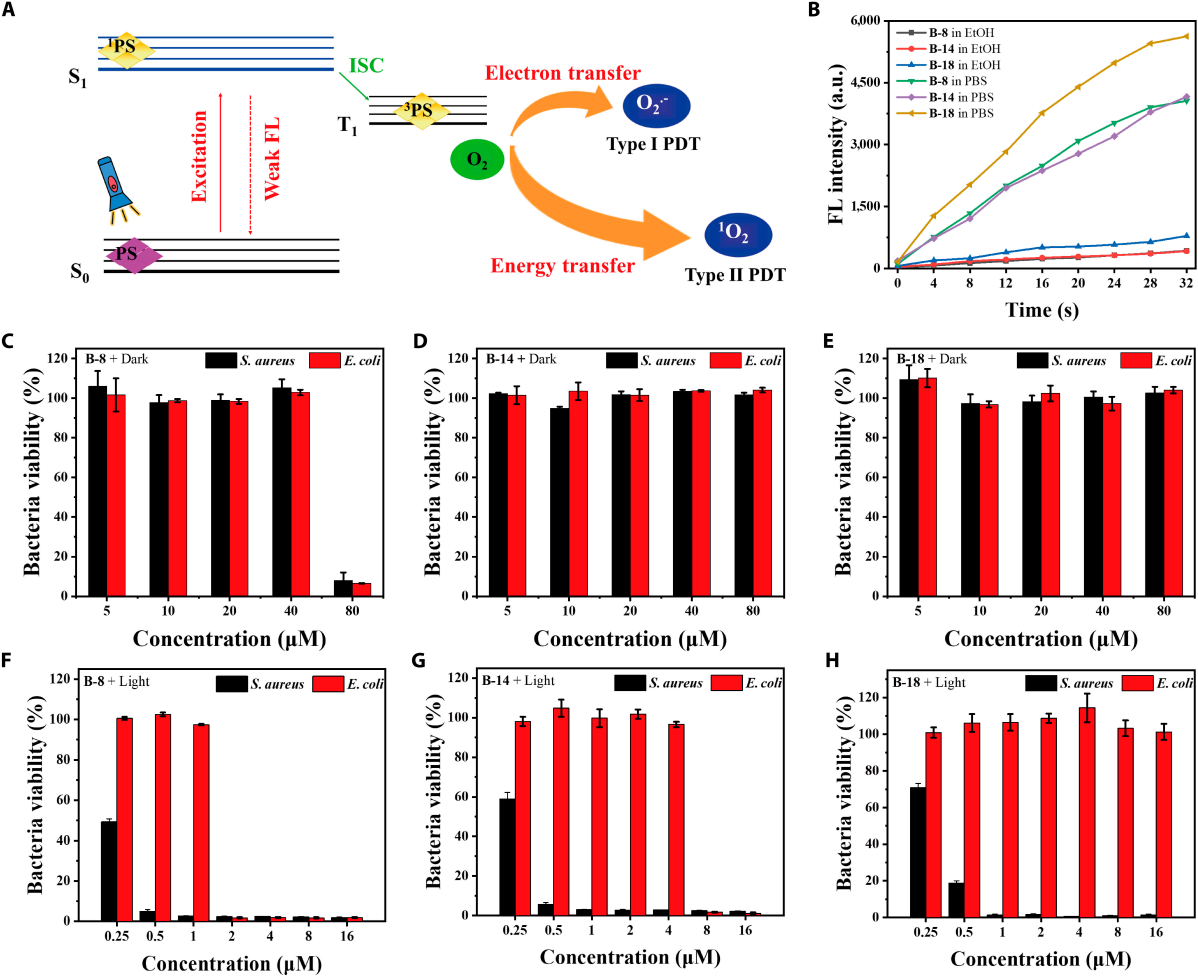
BODIPY derivatives’ ROS generation and MIC detection. (A) Schematic diagram of ROS generation mechanism of PS. (B) Time-course plots of DCFH FL intensity enhancement (concentration: 10 μM). (C to E) MIC of B-8, B-14, and B-18 against bacteria in the dark. (F to H) MIC of B-8, B-14, and B-18 against bacteria with white light irradiation (20 mW·cm^−2^, 30 min).

**Fig. 7.**
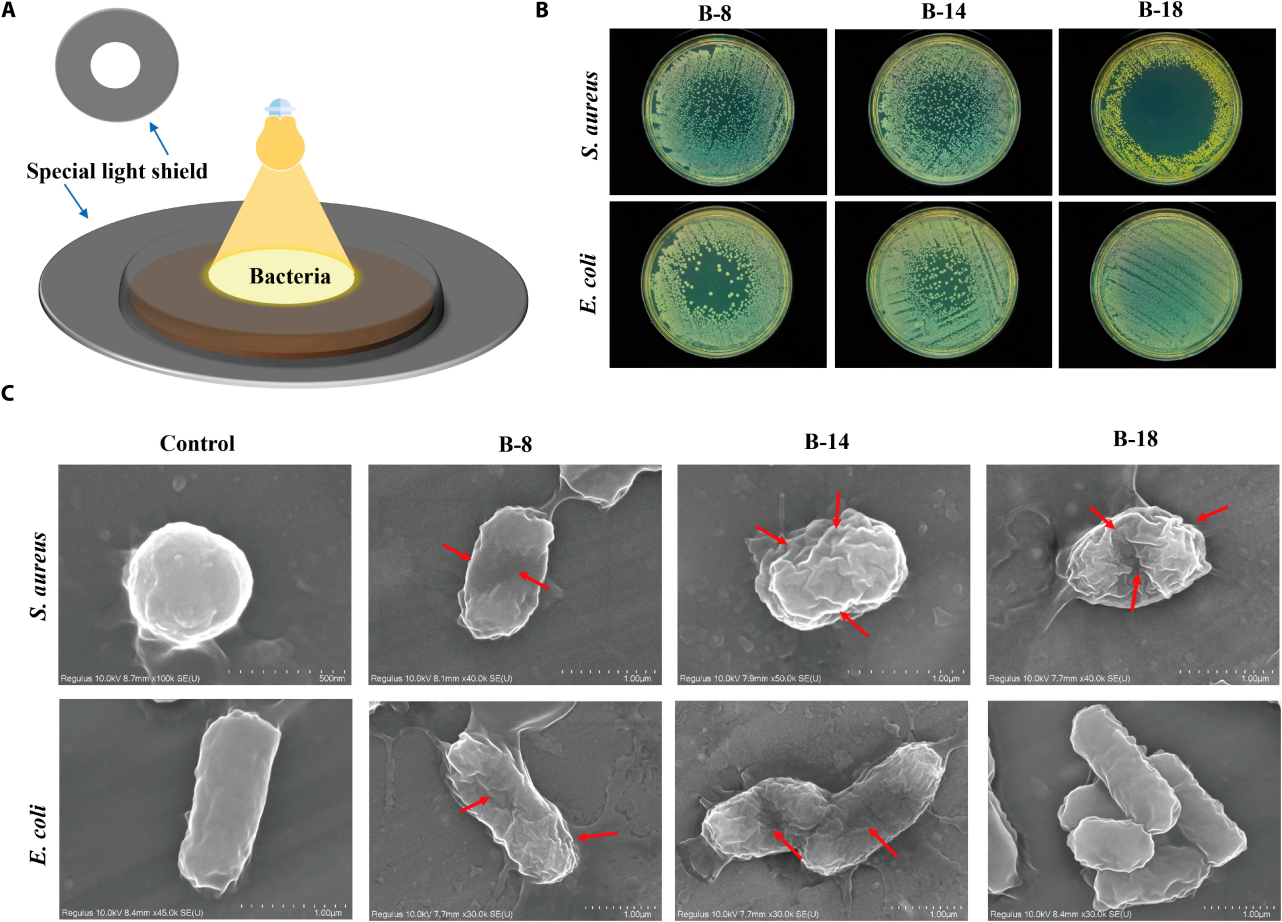
In vitro photodynamic bactericidal efficacy of BODIPY derivatives and morphological analysis of bacterial architecture. (A) Schematic diagram of photodynamic inactivation of bacteria in vitro. (B) B-8, B-14, and B-18 were incubated with *S. aureus* and *E. coli* for 30 min. Uniformly coated on the medium and irradiated with white light for 30 min (20 mW·cm^−2^) and cultured for 24 h away from light. (C) SEM of bacteria after 30-min incubation of compound B-8, B-14, and B-18 with *S. aureus* and *E. coli* with 30-min white light irradiation (20 mW·cm^−2^).

**Fig. 8.**
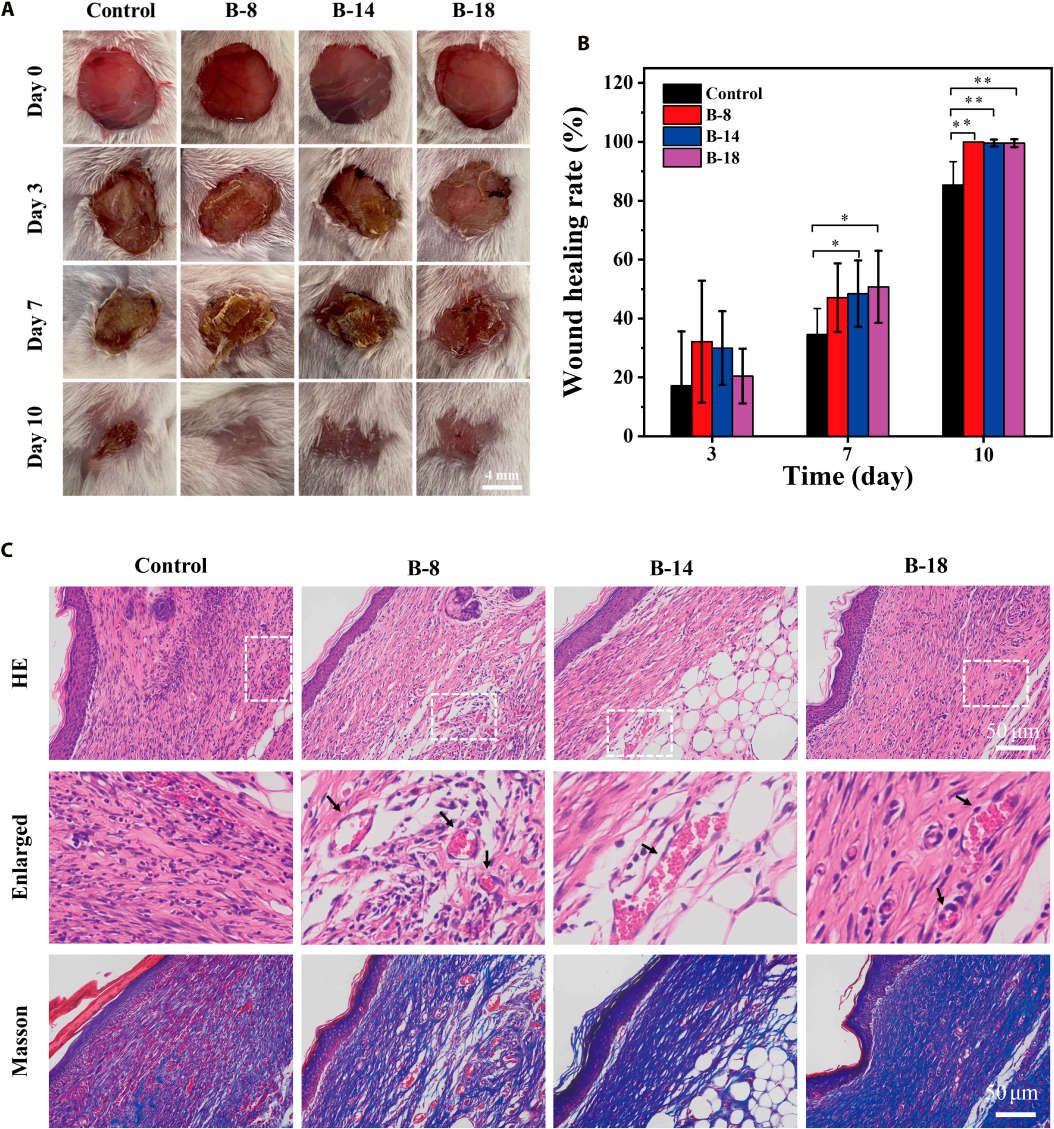
In vivo evaluation of B-8, B-14, and B-18 for the treatment of wounds on mice infected with MRSA. (A) Photos of infected wounds of B-8, B-14, and B-18, and the control group at different periods after 30 min of white light irradiation on day 0. (B) Wound healing ratio of MRSA infection on day 3, day 7, and day 10 (*n* = 3, 20 mW·cm^−2^, *P < 0.05, **P < 0.001). (C) HE and Masson staining of mice’s wound sections on day 10.

Fluorescence spectral analysis and confocal microscopy were employed to characterize the selective interactions of BODIPY derivatives **B-8**, **B-14**, and **B-18** with bacteria. *S. aureus* (gram-positive) and *E. coli* (gram-negative) were chosen to serve as model species. Dead bacterial samples were obtained by disrupting the cell membranes with a 75% ethanol solution. As shown in Fig. 3A and B, **B-8** and **B-14** displayed increased fluorescence emission in the presence of both live and dead bacteria, with dead bacteria showing lower intensities. The diminished fluorescence signal is attributed to the reduced BODIPY derivatives depolymerization in dead bacteria. **B-14** notably exhibited a significant enhancement in emission intensity, approximately 3-fold for *E. coli* and 7-fold for *S. aureus*. Figure 3C shows that **B-18** gave a selective fluorescence response to *S. aureus*, with a 53.2-fold enhancement at 585 nm. This specificity maybe attributed to the disassembly of **B-18** aggregate by inducing with the live gram-positive bacteria, which was not observed in dead bacteria or gram-negative species. The distinct structural and compositional properties of gram-negative and gram-positive bacterial cell walls, characterized by variations in surface charge and hydrophobicity, are pivotal for the differential assembly and disassembly mechanisms of these species [27–29]. These aggregates exhibit a tendency for selective dispersion in response to bacterial cell wall variations.

Confocal laser scanning microscopy was utilized to image both live and dead bacterial samples after verifying the selective staining capabilities of the BODIPY derivatives. Sytox Deep Red, a nucleic acid stain specific for compromised cell membranes, was used in conjunction with the BODIPY derivatives. Emissions were recorded within 530 to 560 nm for **B-8**, **B-14**, and **B-18** (green channel) and 650 to 750 nm for Sytox Deep Red (red channel). **B-8** fluorescence was observed for both live and deceased *E. coli* and *S. aureus*, albeit requiring dye removal for clarity. **B-14** showed staining efficacy comparable to **B-8** with a reduced background signal, highlighting its potential for wash-free imaging (Fig. S14 and Fig. 3C). In contrast, **B-18** selectively emitted fluorescence upon interaction with *S. aureus*, with minimal signal detected for *E. coli* and dead *S. aureus* (Fig. 3F). Notably, **B-18** effectively stained *S. aureus* within a 10-min incubation time (Fig. S15). These results highlight the utility of **B-8** and **B-14** in costaining with Sytox Deep Red for viability assessment, while **B-18** serves as a selective marker for gram-positive bacteria. Zeta potential data were tested to evaluate the electrostatic interactions between bacterial strains and the BODIPY compounds. Figure 3D shows that *S. aureus* exhibited a positive zeta potential shift of approximately 3 mV upon interaction with **B-8**, **B-14**, or **B-18**, suggesting successful binding to the bacteria. **B-8** induced a more significant shift in *E. coli* compared to **B-14** and **B-18**, with **B-18** causing only a minor change, indicating that electrostatic forces alone are insufficient to disassemble **B-18** aggregates.

The ability of our approach to discriminate between Gram types was further demonstrated using a panel of 8 bacterial pathogens, including gram-negative strains (*S. enteritidis*, *P. aeruginosa*, *P. vulgaris*, and *E. coli*) and gram-positive strains (*E. faecalis*, *L. monocytogenes*, *B. cereus*, and *S. aureus*). **B-8** effectively stained both gram-positive and gram-negative strains, with improved image clarity after washing (Fig. S16). **B-14** showed similar imaging capabilities to **B-8** but in a wash-free manner (Fig. 4A). In contrast, **B-18** displayed bright fluorescence upon interaction with gram-positive strains, with minimal signal from gram-negative strains (Fig. 4B), confirming again its high selectivity for gram-positive bacteria.

**B-18** has shown remarkable performance in rapid Gram typing and bacterial viability detection. In costaining experiments with **B-18**, gram-positive *S. aureus* exhibited distinct green fluorescence, contrasting with the non-fluorescent gram-negative *E. coli* (Fig. 5A). A positive correlation between *S. aureus* concentration and fluorescence intensity was observed under UV irradiation within the range of 5 × 10^5^ to 1 × 10^9^ CFU/ml. A regression analysis provided a fitting curve: *y* = 17.8738*x* − 96.8693, with an *R*^2^ value of 0.9902, which is beneficial for developing sensors for rapid detection of *S. aureus* concentration (Fig. S17). After confirming the differential marking capabilities for live and dead bacteria, we used **B-18** to monitor vitality changes in *S. aureus* treated with vancomycin, a gram-positive specific antibiotic. A gradual decline in fluorescence intensity with increasing vancomycin exposure (Fig. 5B) indicates the vitality change of gram-positive bacteria under antibiotic stress. These data demonstrated that **B-18** could serve as a valuable tool for real-time antibiotic efficacy evaluation.

### Photodynamic inactivation of bacteria in vitro

PDT utilizes PSs to generate ROS through Type I or II mechanisms, as shown in Fig. 6A [30]. Our previous research has confirmed that the long-chain-tailed BODIPYs aggregates were a novel class of heavy-atom-free PSs [31]. Thus, we were eager to investigate their behavior on the photodynamic inactivation to various bacterial species.

Firstly, we examined the generation of ROS in various BODIPY derivatives, employing **DCFH** as an indicator for ROS production. As illustrated in Fig. 6B and Fig. S18, the results indicate BODIPY aggregates in PBS exhibit a significant increase in ROS generation under 20 mW·cm^-2^ irradiation, whereas their monomers in EtOH display reduced ROS generation under identical conditions. Notably, **B-18** demonstrated superior ROS generation capabilities compared to **B-8** and **B-14**, in both aggregated and monomeric states. Aggregates, which limit molecular motion and affect the intersystem crossing process, were observed to produce approximately an order of magnitude more ROS than monomers [32]. These findings suggest that the aggregated state of the BODIPY compounds is particularly effective for ROS production.

We nest assessed their antibacterial efficacy against *S. aureus* and *E. coli* using the broth microdilution, light irradiation plate methods, and SEM. As depicted in Fig. 6C to E, our datarevealed that **B-8** showed a remarkable dark toxicity at the concentration over 80 μM, **B-14** and **B-18** exhibit little dark toxicity even at the concentration as high as 80 μM. This discrepancy is attributed to the medium-length alkyl chains **B-8**, which may enhance bacterial cell permeability, allowing **B-8** to penetrate and inactivate bacteria [33].

As shown in Fig. 6F to H, these compounds at 0.25 μM effectively inhibit *S. aureus* growth under the white light irradiation. The minimal inhibitory concentration (MIC) was determined to be 1 μM for **B-8** and **B-14** against *S. aureus* and 2 μM for **B-8** and 8 μM for **B-14** against *E. coli*. **B-18** demonstrated an MIC of 1 μM against *S. aureus*, with a less pronounced effect on *E. coli*. The superior antibacterial activity against *S. aureus* is likely due to the thicker peptidoglycan layer in gram-positive bacteria, facilitating compound anchoring and ROS delivery, thereby amplifying the killing radius and plasma membrane disruption.

To mitigate the elevated ROS production of compound aggregates in a liquid environment, we evaluated the photodynamic inactivation potential of compounds when anchored to the bacterial surface. Employing a light irradiation plate method, we further examined the photodynamic inactivation capabilities of BODIPY derivatives. As shown in Fig. 7A, with the exception of a central circular area that was left exposed to white light, a significant reduction was observed in *S. aureus* within the irradiated zone of the **B-8** group, while the dark area showed dense bacterial growth. A similar pattern was observed for *E. coli*, with a substantial decrease in the number of bacteria under the light-exposed area in comparison with the sparse and compact growth in the dark area. Notably, the **B-18** group demonstrated almost 100% inhibition of *S. aureus* growth in the light-exposed area, in stark contrast to both the dark area and the *E. coli* group (Fig. 7B).

To gain more insights into the antibacterial mechanism of these BODIPY compounds, we set to visualize the bacteria morphological changes under white light irradiation. The control group of *S. aureus* exhibited the quintessential smooth, rounded cocci morphology, as depicted in Fig. 7C. In contrast, upon exposure to **B-8**, **B-14**, and **B-18**, followed by irradiation, the cell membranes of *S. aureus* underwent significant disruption, with a pronounced transition to an oval configuration, highlighted by red arrows. *E. coli*, characterized by a rod-shaped and smooth surface in the untreated state, displayed substantial morphological damage upon treatment with **B-8** and **B-14** under white light, with more pronounced effects relative to those observed with **B-18**. This damage encompassed cell membrane rupture and a loss of cell wall integrity, culminating in cellular shrinkage, as indicated by red arrows. Collectively, these observations indicate that **B-8** and **B-14** are potent in disrupting bacterial cell wall structures under light irradiation, while **B-18** appears to selectively target the cell wall of gram-positive bacteria. The antibacterial efficacy of these BODIPY derivatives is mainly attributed to the inactivation of bacteria through ROS produced by exposure to white light.

### In vivo infection assay

Following the demonstration of antibacterial effects for **B-8**, **B-14**, and **B-18** in vitro, we proceeded to assess their efficacy in an in vivo setting. Utilizing a murine model of MRSA skin infection, we established MRSA-infected wounds on the dorsal skin of BALB/c mice. The infected mice were randomly allocated into 4 groups and designated as follows: PBS (control group), **B-8**, **B-14**, and **B-18**. Each group underwent irradiation at an intensity of 20 mW·cm^-2^ for 30 min on day 0.

To monitor wound healing progression, photographic documentation was taken at various time points (as depicted in Fig. 8A). Initially, on day 3, no significant differences in wound healing were observed across all groups. By day 7, the **B-8**, **B-14**, and **B-18** groups exhibited a general trend of enhanced wound healing compared to the control group. On day 10, the formation of new skin tissue was evident in the **B-8**, **B-14**, and **B-18** groups, with a significantly accelerated wound healing rate compared to the control group (as shown in Fig. 8B). For histological analysis, wound tissue sections from mice sacrificed on day 10 were stained with HE and Masson trichrome. The HE staining revealed the presence of new blood vessels beneath the neogenic skin in the treatment groups (Fig. 8C). Additionally, Masson staining delineated the structured collagen fibers in the healed tissue of the treated groups. Collectively, the in vivo findings reinforce the strong antibacterial activity of **B-8**, **B-14**, and **B-18** against MRSA under light irradiation and their significant contribution to the wound healing process, aligning well with our in vitro observations.

## Discussion

Our study introduces a pioneering approach to the identification and selective photodynamic inactivation of bacteria using BODIPY derivatives featuring distinct alkyl chain lengths. The photophysical properties and bacterial recognition capabilities of these compounds—**B-8**, **B-14**, and **B-18**—have unveiled subtleties in their interactions with bacterial cells, culminating in varied efficacies in photodynamic inactivation.

The length of the alkyl chains in the BODIPY derivatives has a pronounced effect on their solubility and aggregation behavior, which in turn dictates their photophysical characteristics. **B-14** and **B-18**, in particular, exhibited a notable ACQ effect in aqueous environments, suggesting a propensity for forming aggregates that significantly influence fluorescence intensity. This behavior is likely driven by hydrophobic interactions among the alkyl chains, and further investigation is warranted to understand how these interactions affect the balance between self-assembly and cellular interaction. The selectivity of the BODIPY derivatives for different bacterial species is attributed to the structural and compositional heterogeneity of bacterial cell walls [27–29]. **B-18** demonstrated exceptional selectivity for gram-positive bacteria, potentially due to its ability to selectively label viable cells, leading to a substantial fluorescence enhancement. This selectivity may result from specific interactions between **B-18**’s alkyl chains and the peptidoglycan layer of gram-positive bacteria, a mechanism that deserves deeper exploration. The photodynamic inactivation efficacy of the BODIPY derivatives varied with alkyl chain length, with **B-18** showing the highest potency against gram-positive bacteria, especially *S. aureus*. This superior activity may be due to the synergistic effect of selective bacterial recognition and the photophysical properties of **B-18**, facilitating efficient energy transfer and ROS generation upon light irradiation.

Despite the promising results, our study has limitations that open avenues for future research. Elucidating the precise mechanisms by which the self-assembly of BODIPY derivatives influences their interactions with bacterial cells and their photodynamic effects is essential. Future studies should concentrate on the molecular dynamics of these interactions and the influence of the self-assembly process on them. Additionally, optimizing the self-assembly behavior of the BODIPY derivatives to achieve orderly and tunable assembly, without compromising their photophysical properties and bacterial recognition capabilities, is a promising direction. Such optimization could lead to the development of a new class of antimicrobial agents with broad-spectrum efficacy, capable of precisely targeting and eliminating bacteria in various systemic infections, including those in challenging environments like the urinary tract or bloodstream.

In conclusion, this study presents long-chain-tailed BODIPY derivatives as a dual-modality tool for bacterial identification and elimination. The compounds **B-8**, **B-14**, and **B-18** have distinct interactions with bacterial surfaces, with **B-8** capable of staining both live and dead bacteria, **B-14** providing wash-free staining, and **B-18** showing selective fluorescence enhancement with live gram-positive bacteria. The 53.2-fold fluorescence increase observed with **B-18** upon interaction with *S. aureus*, along with its potent photodynamic antibacterial activity at 1 μM, indicates its therapeutic potential in in vivo MRSA wound infection models. This work offers innovative perspectives in bacterial imaging and therapy, possibly revolutionizing our approach to combating antibiotic resistance.

## Data Availability

All presented data in this manuscript and supporting information are available from the corresponding authors upon reasonable request.
