# Supplementary material for: Wash-Free Bacterial Gram-Typing and Photodynamic Inactivation with Long-Chain-Tailed BODIPY Derivatives
Source: Biomater Res. 2024 Sep 3;28:0069. doi: 10.34133/bmr.0069 (PMC11370751; doi:10.34133/bmr.0069)
Supplement: Supplementary 1 — Figs. S1 to S18 Table S1 [file bmr.0069.f1.docx]

**Supplementray information**

Wash-free Bacterial Gram-Typing and Photodynamic Inactivation with Long-chain-tailed BODIPY Derivatives

Yuefeng Ji, Jigai Li, Chunping Chen, Chunxiang Piao*, Xin Zhou*, Juyoung Yoon*

*Corresponding author:

Chunxiang Piao

e-mail: cxpiao@ybu.edu.cn

Xin Zhou

e-mail: zhouxin@qdu.edu.cn

Juyoung Yoon

e-mail: jyoon@ewha.ac.kr

Figure S1. Synthetic route of **B-8**, **B-14** and **B-18**.


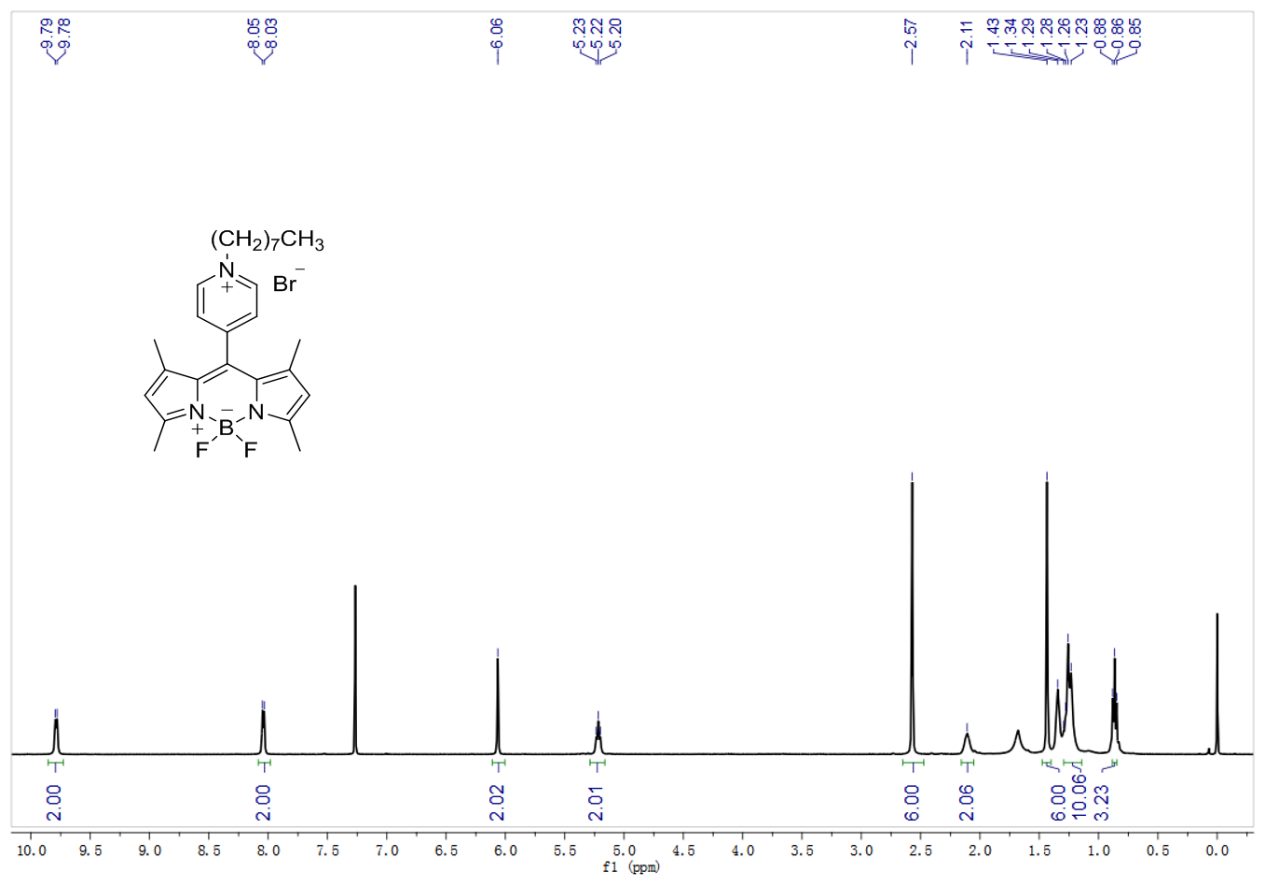


Figure S2. ^1^H NMR spectrum of compound **B-8** in CDCl_3_.


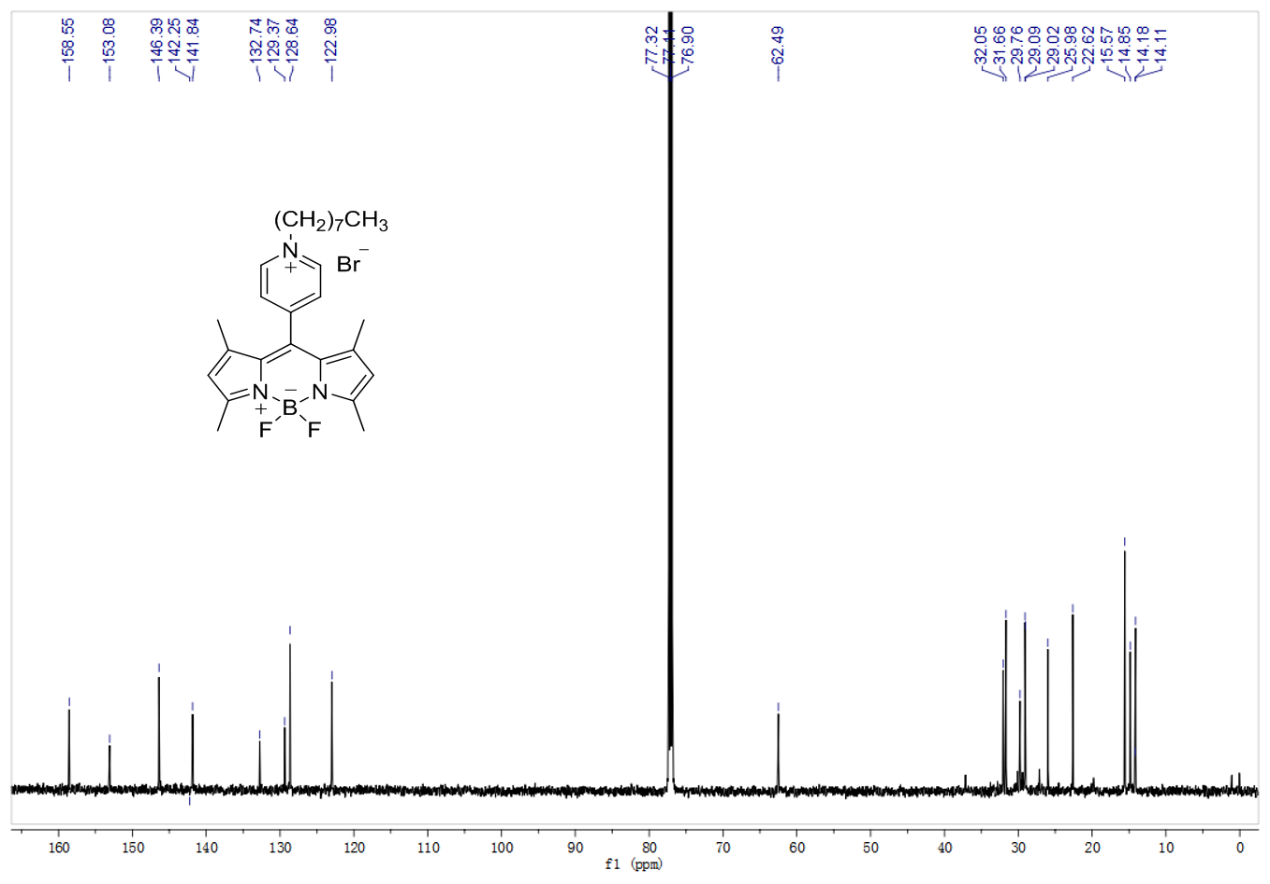


Figure S3. ^13^C NMR spectrum of compound **B-8** in CDCl_3_.


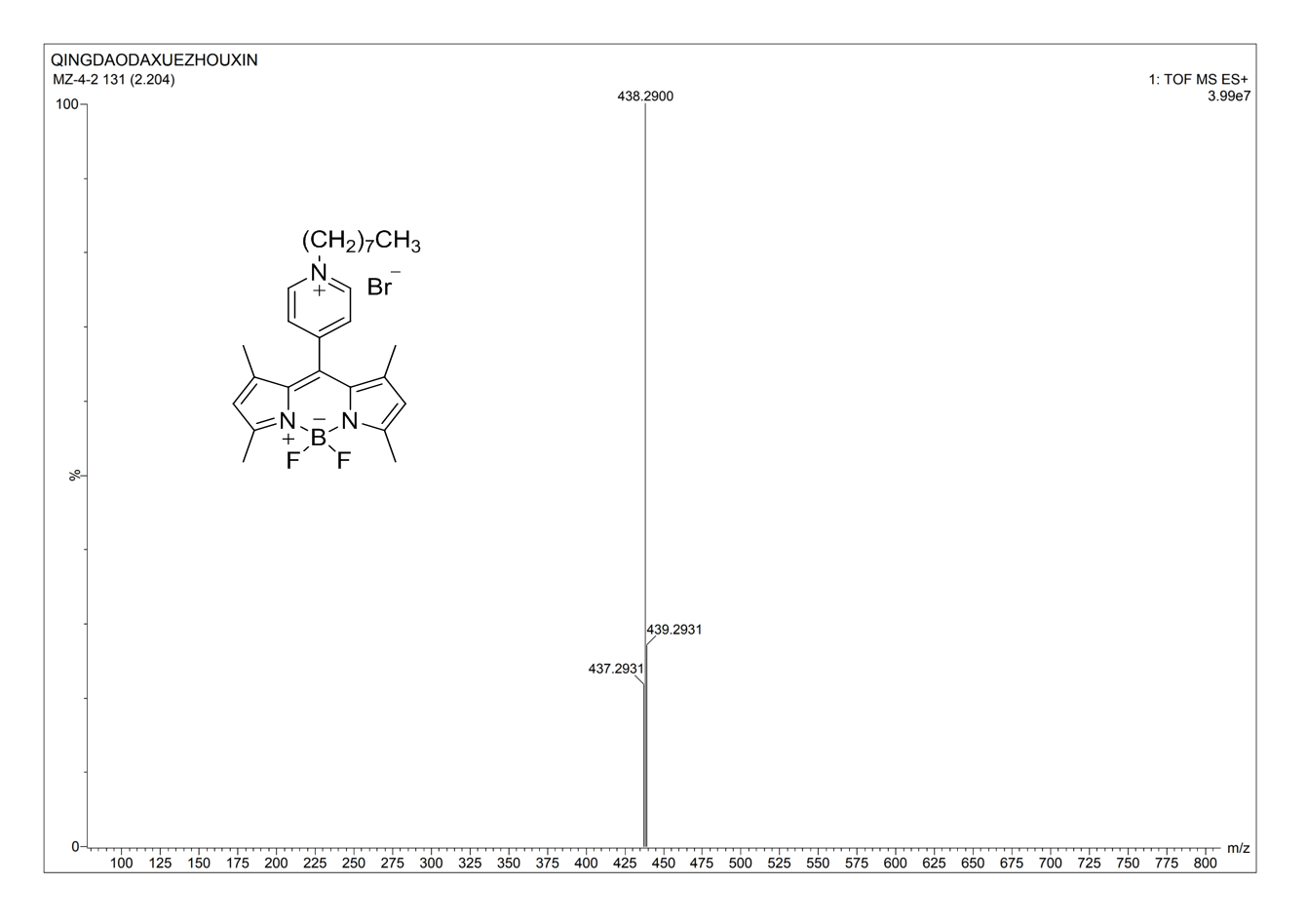


Figure S4. HRMS spectrum of compound **B-8**.


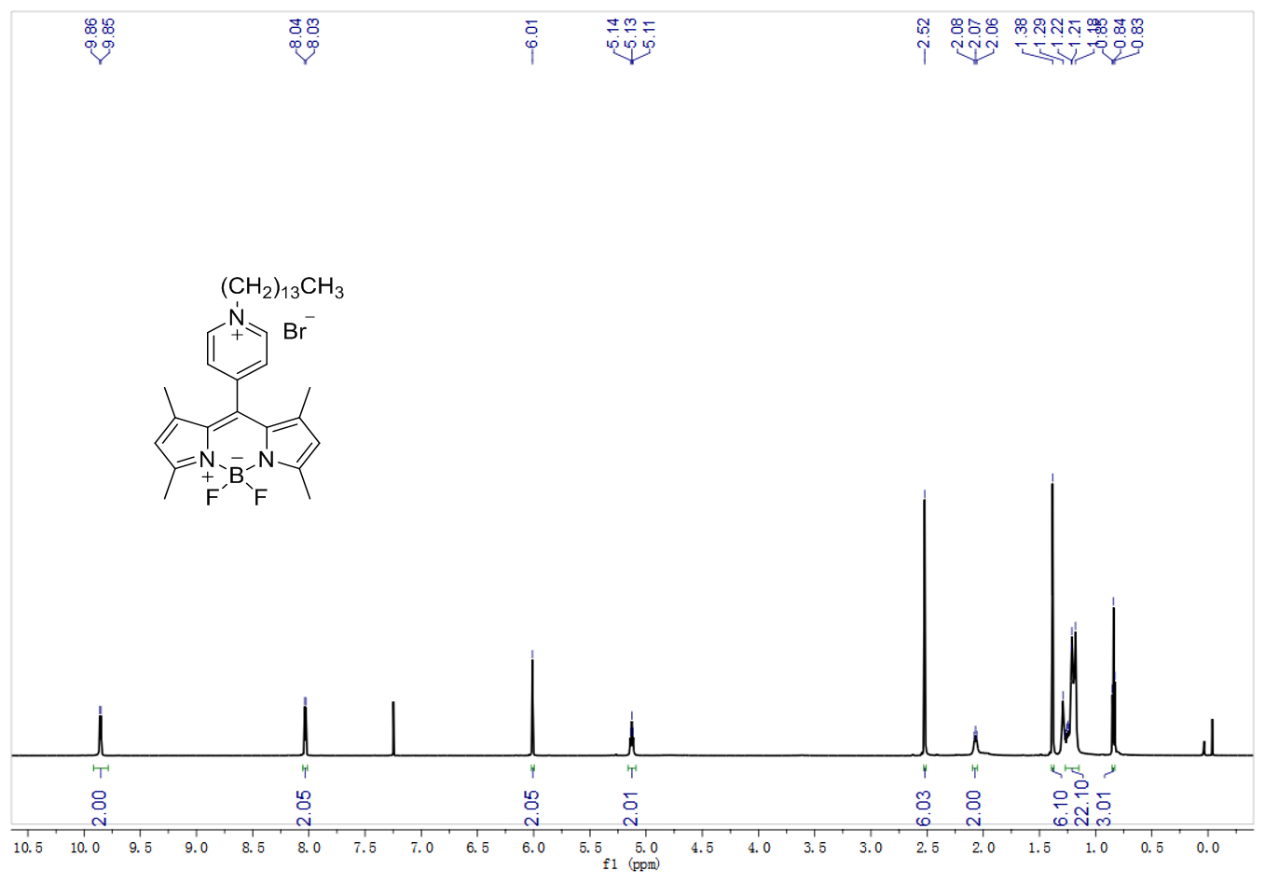


Figure S5. ^1^H NMR spectrum of compound **B-14** in CDCl_3_.


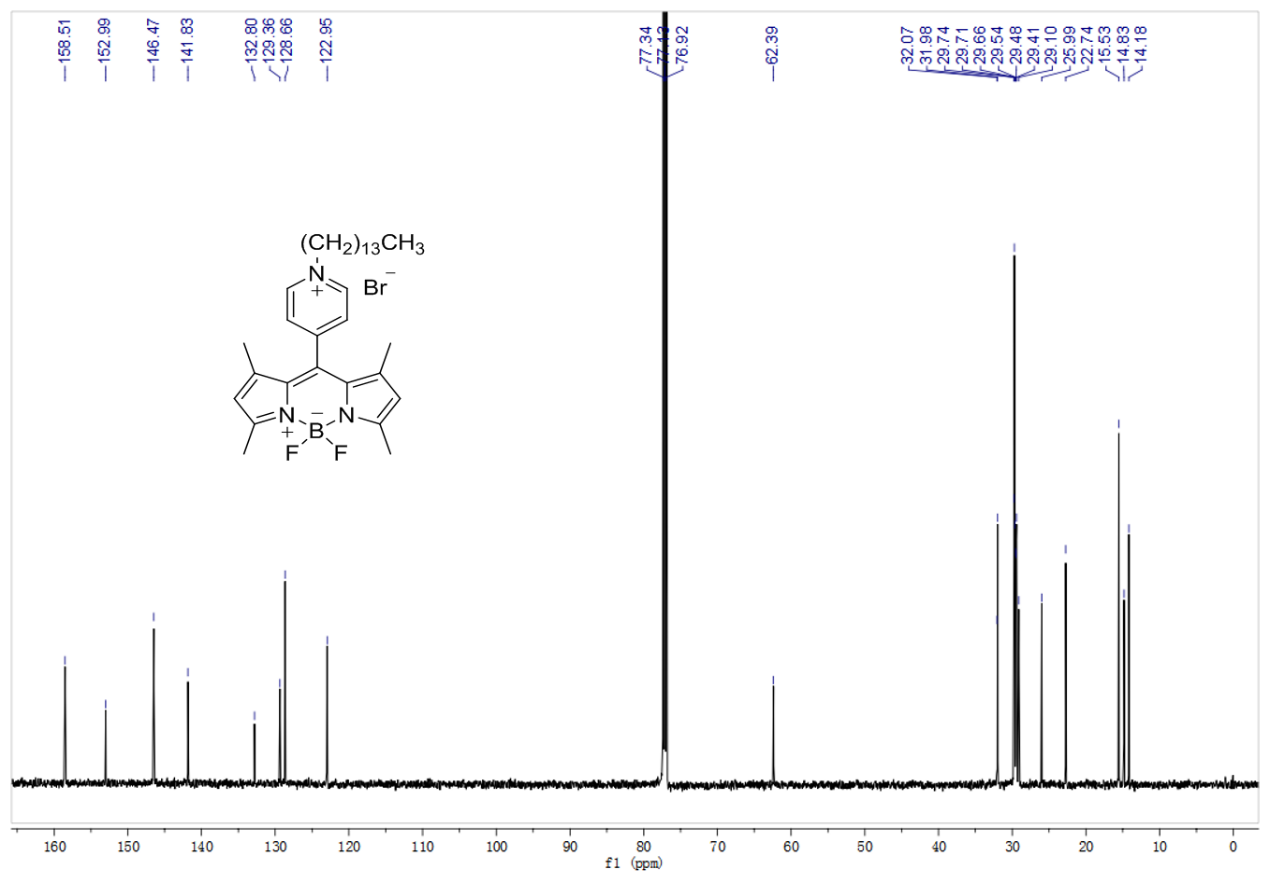


Figure S6. ^13^C NMR spectrum of compound **B-14** in CDCl_3_.


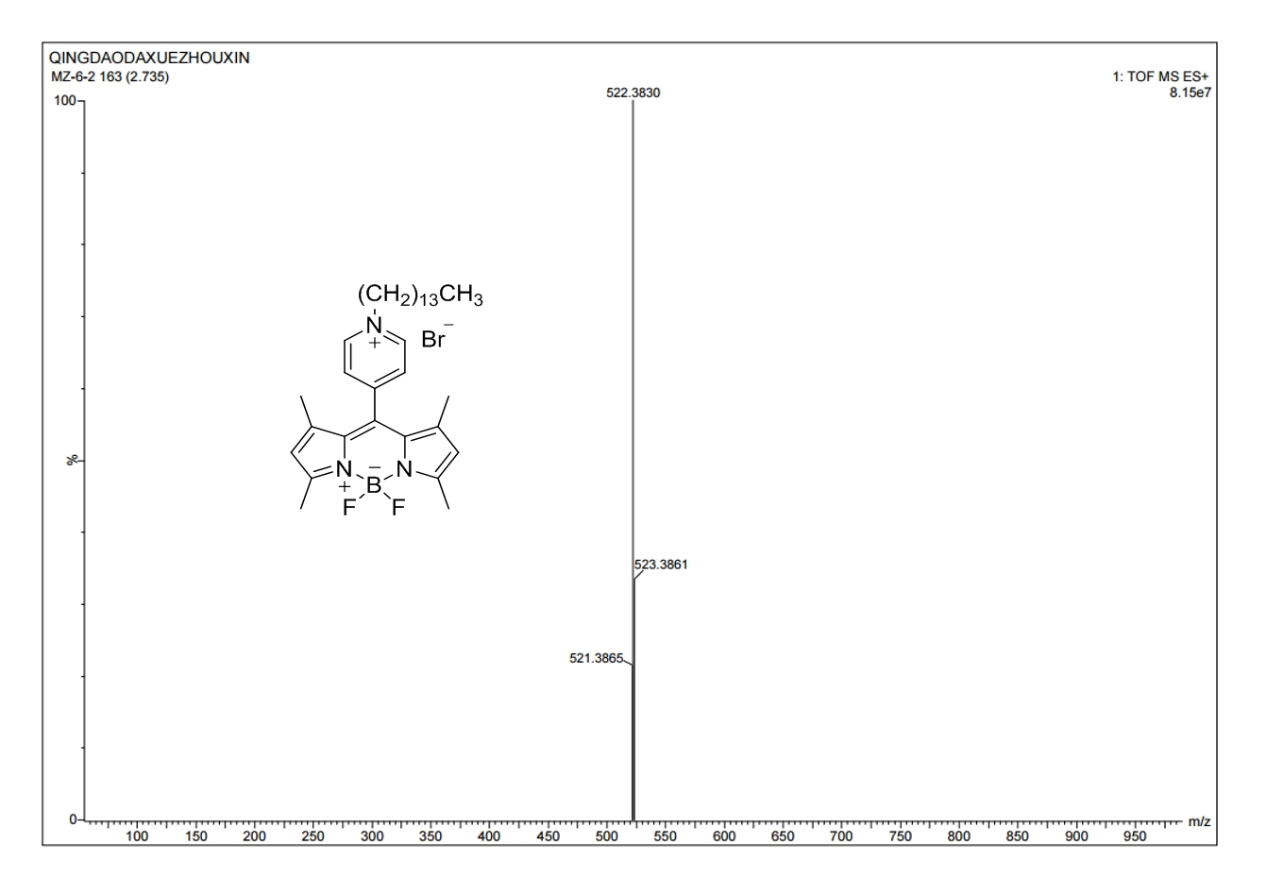


Figure S7. HRMS spectrum of compound **B-14**.


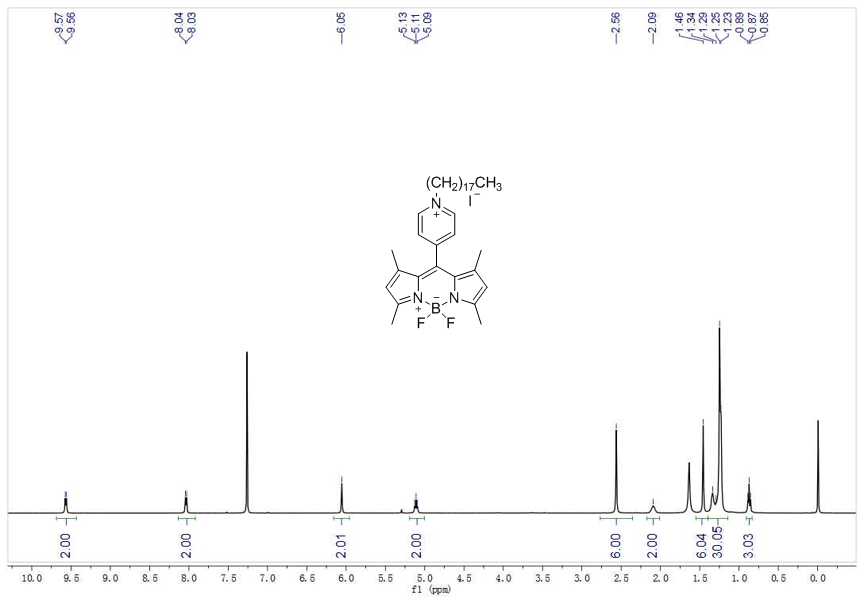


Figure S8. ^1^H NMR spectrum of compound **B-18** in CDCl_3_.

Figure S9. ^13^C NMR spectrum of compound **B-18** in CDCl_3_.


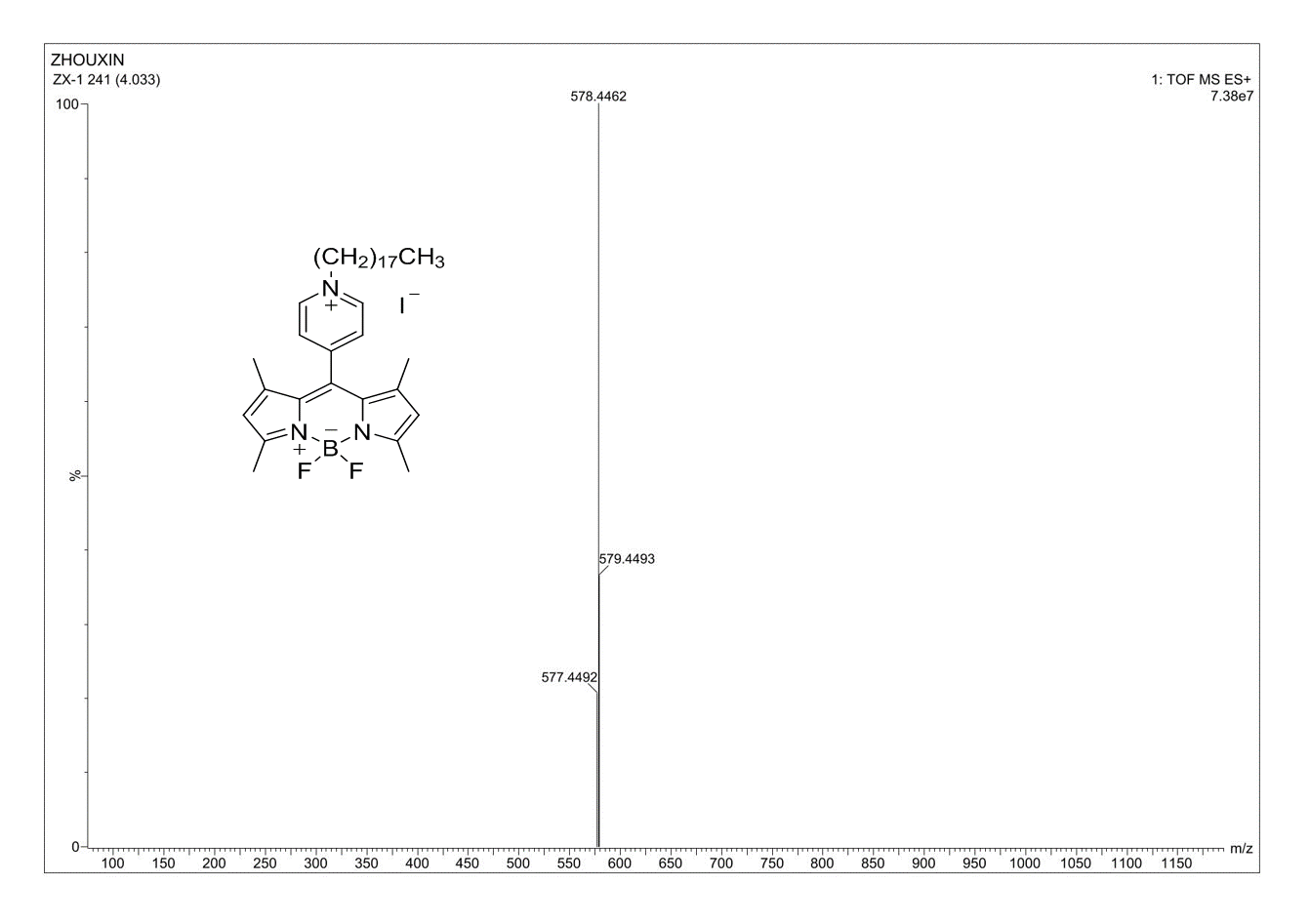


Figure S10. HRMS spectrum of compound **B-18**.


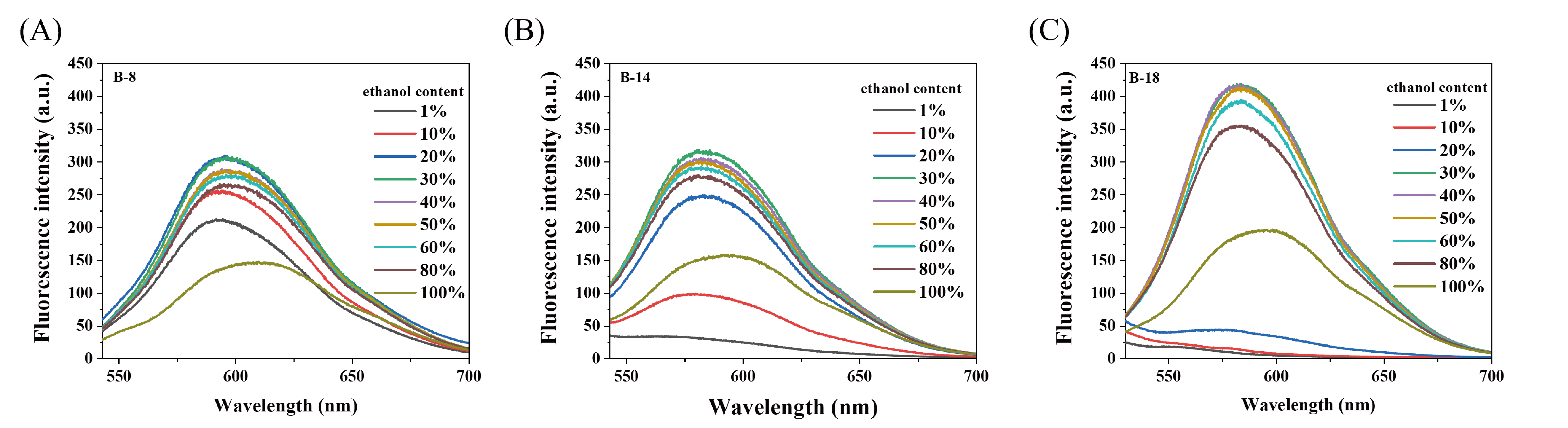


Figure S11. Fluorescent spectra of (A) **B-8**, (B) **B-14,** and (C) **B-18** in a mixture of EtOH and PBS. (concentration: 10 μM)


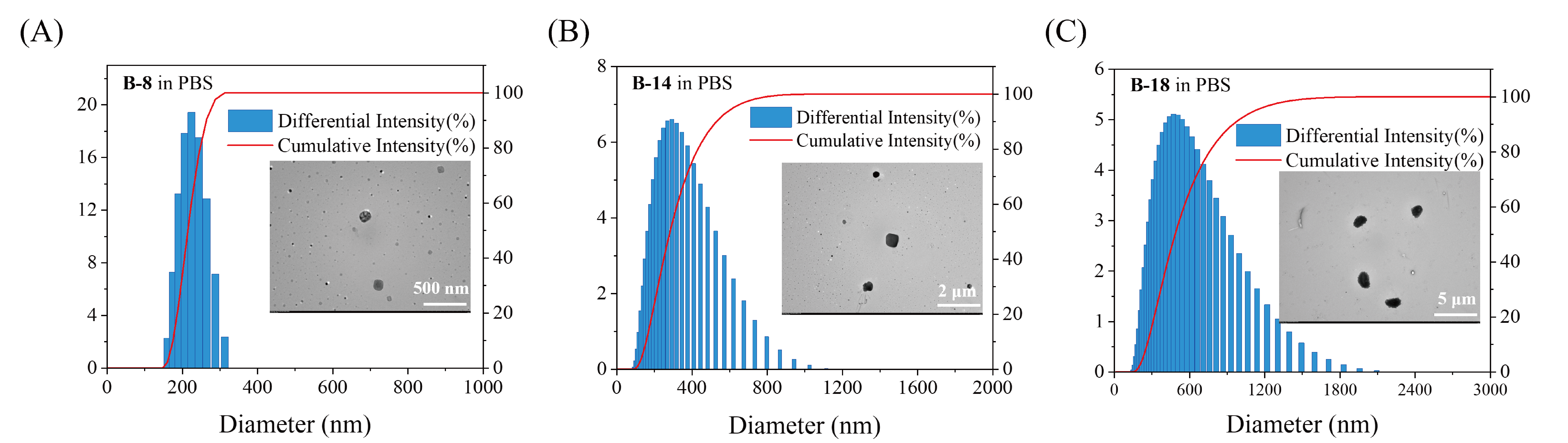


Figure S12. DLS and TEM image (inset) of the aggregates of (A) **B-8**, (B) **B-14**, and (C) **B-18** in PBS.


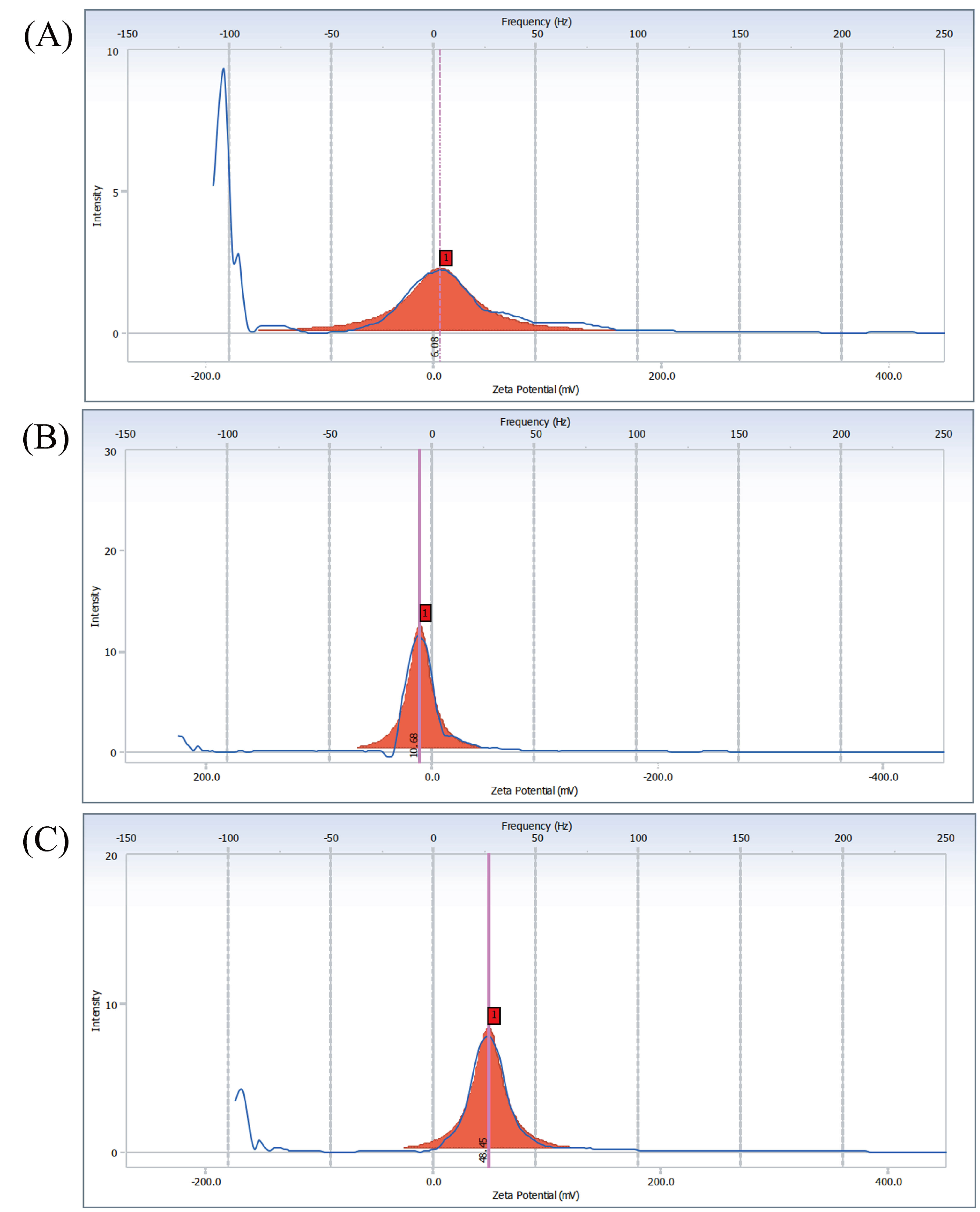


Figure S13. Zeta potential of (A) **B-8**, (B) **B-14** and (C) **B-18** in aqueous solution. (concentration: 10 μM)


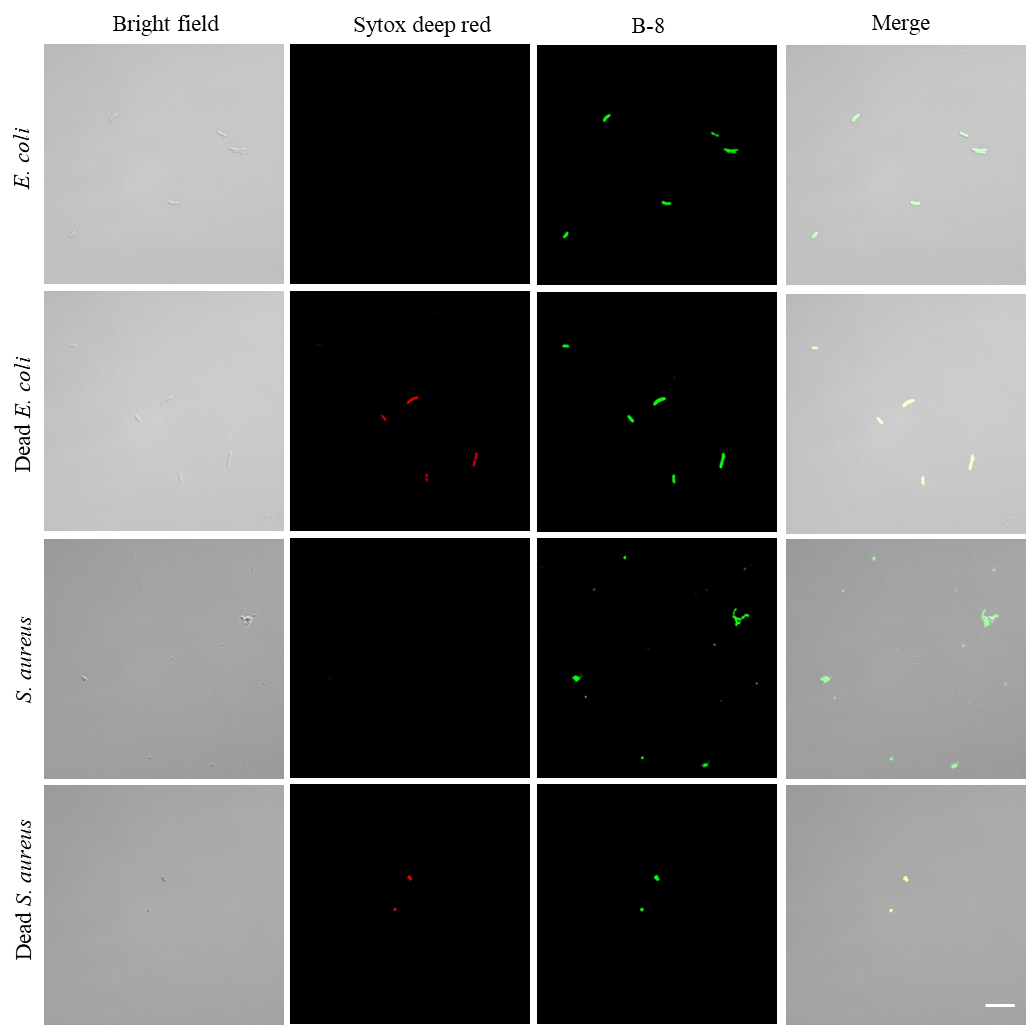


Figure S14. CLSM imaging of live/dead *S. aureus* and *E. coli* co-stained with **Sytox Deep Red** and **B-8** (concentration: 10 μM, scale: 5 μm, **B-8**: Ex: 515 nm, Em: 530-560 nm, **Sytox Deep Red**: Ex: 635 nm, Em: 650-750 nm).


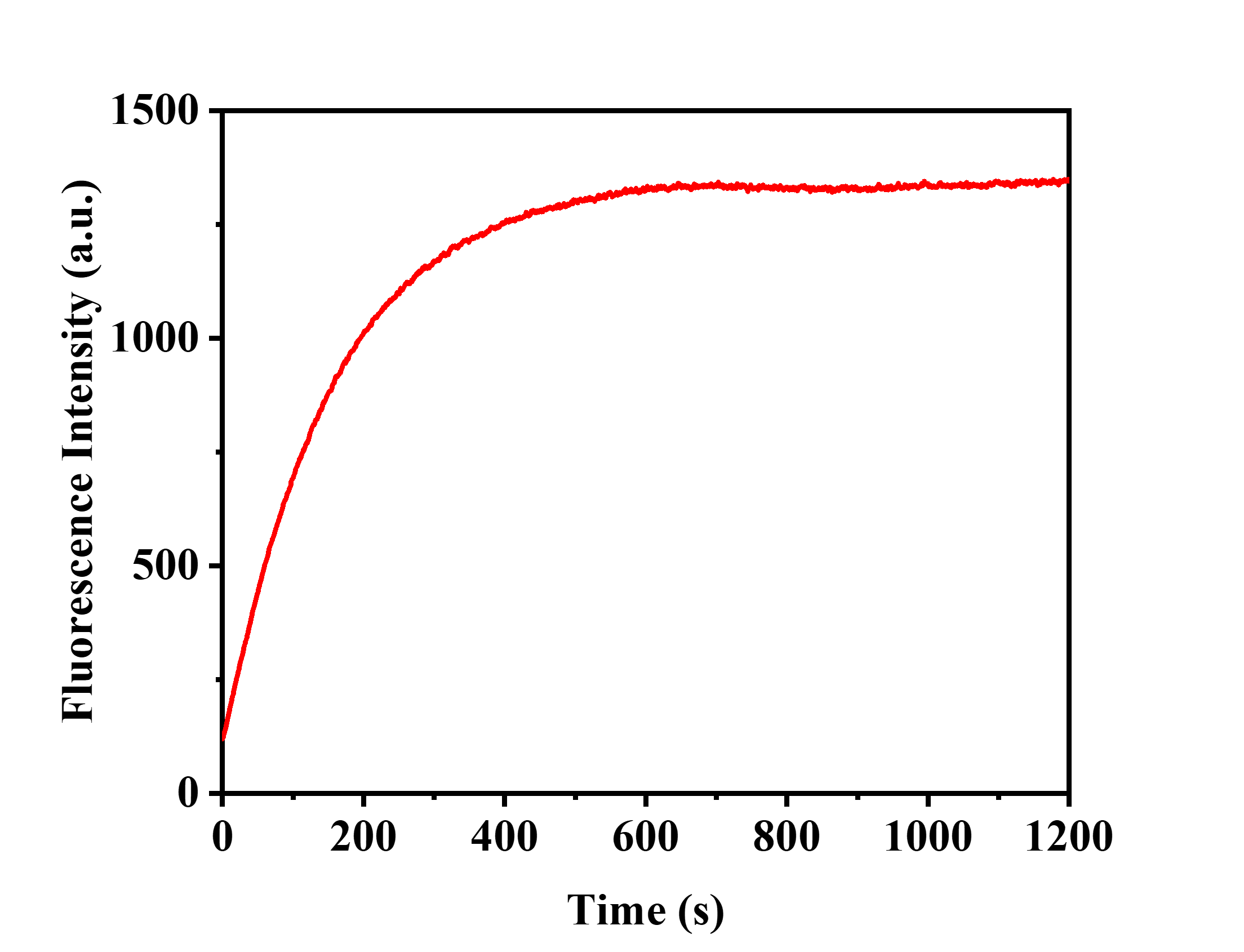


Figure S15. Fluorescence intensity-time profile of *S. aureus* following co-incubation with **B-18**.


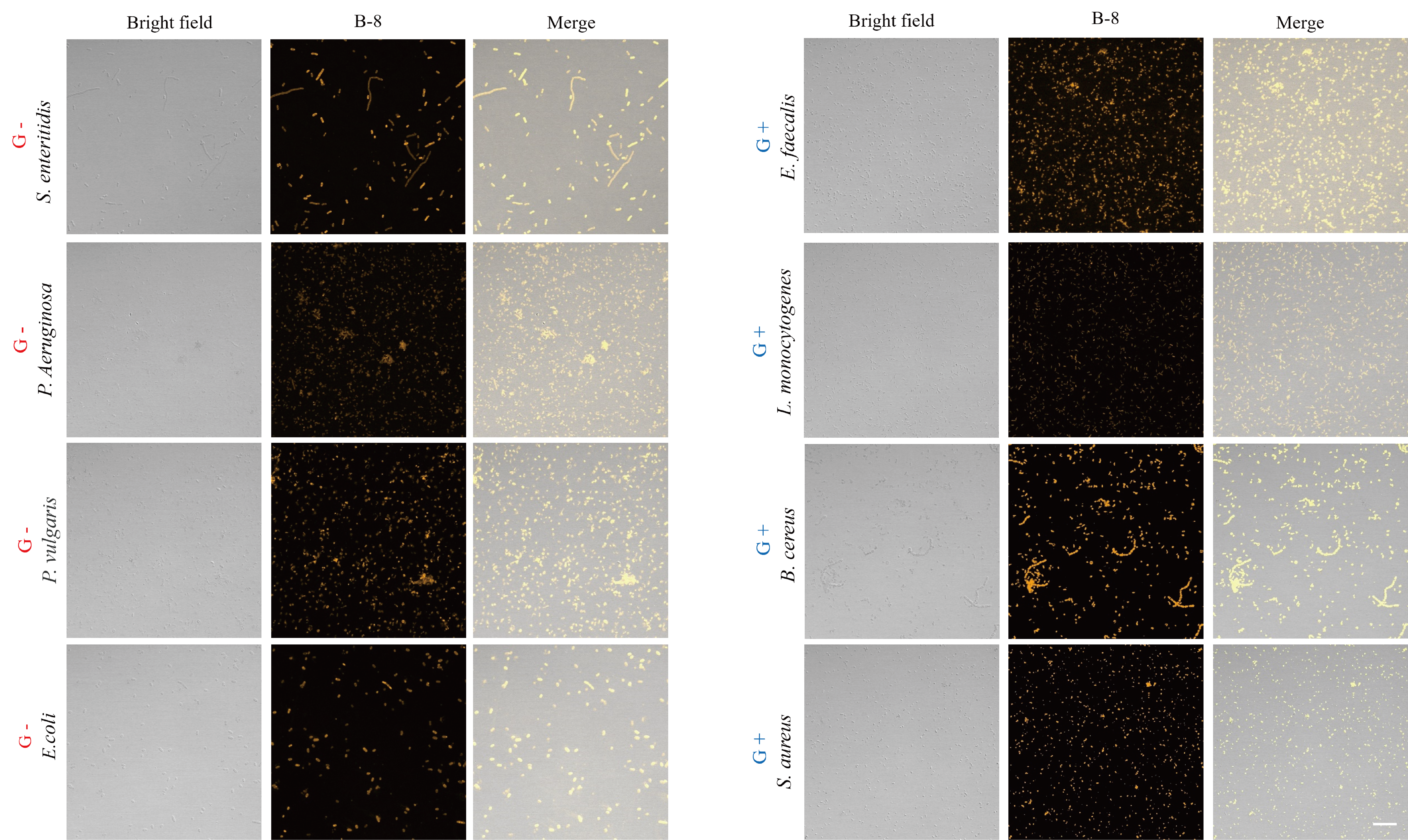


Figure S16. CLSM imaging of **B-8** with four Gram-negative bacteria (*S. enteritidis*, *P. aeruginosa*, *P. vulgaris Hauser,* and *E. coli*) and four Gram-positive bacteria (*E. faecalis, L. monocytogenes, B. cereus,* and *S. aureus*). (concentration: 10 μM, scale bar: 10 μm, Ex: 515 nm, Em: 550-650 nm)


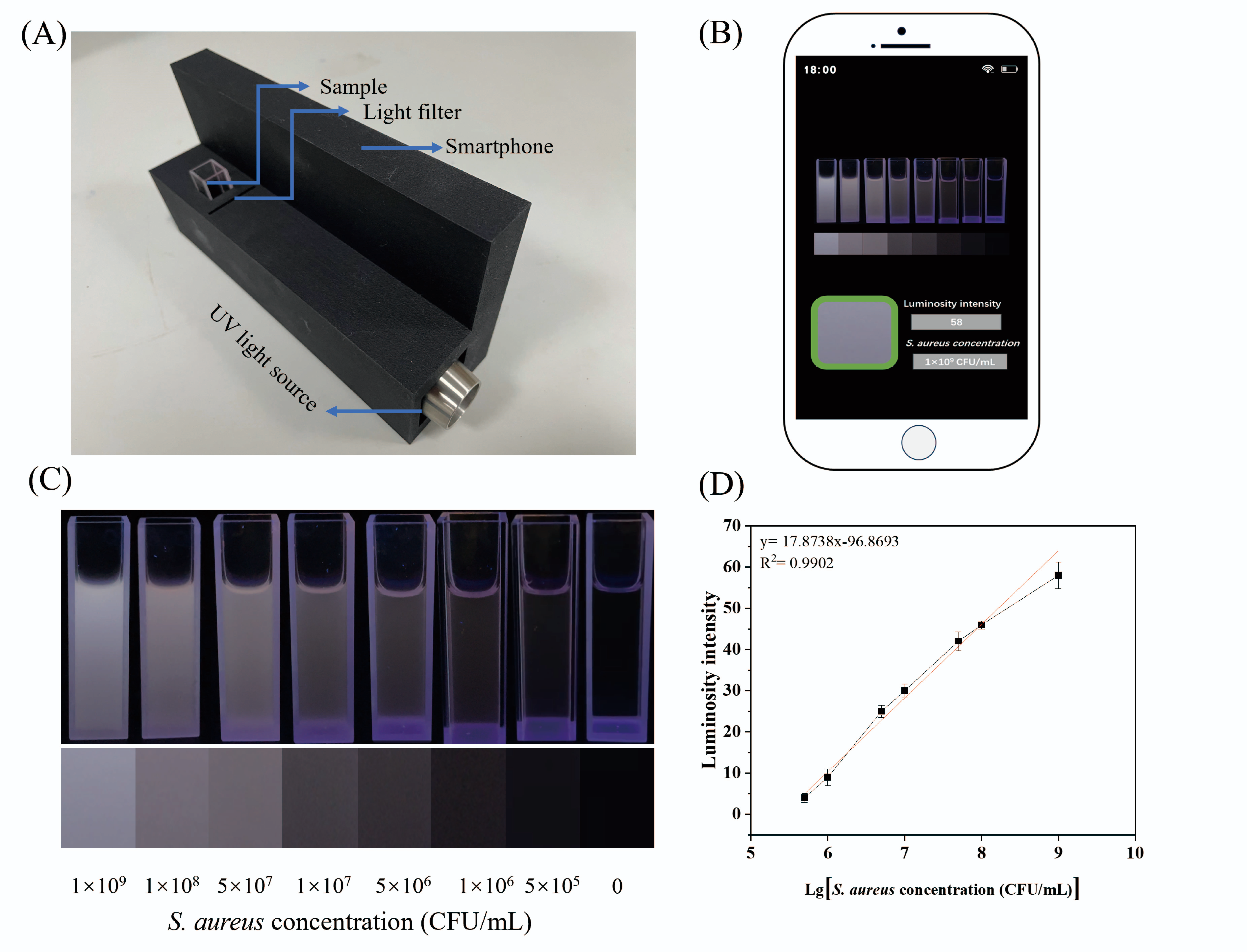


Figure S17. (A) Actual photo of the fluorescent sensor.(B) Schematic diagram of smartphone detection. (C)Under UV light illumination, *S. aureus* concentration was observed with the naked eye using **B-18**. (D)Linear correlation between luminosity intensity and *S. aureus* concentration.


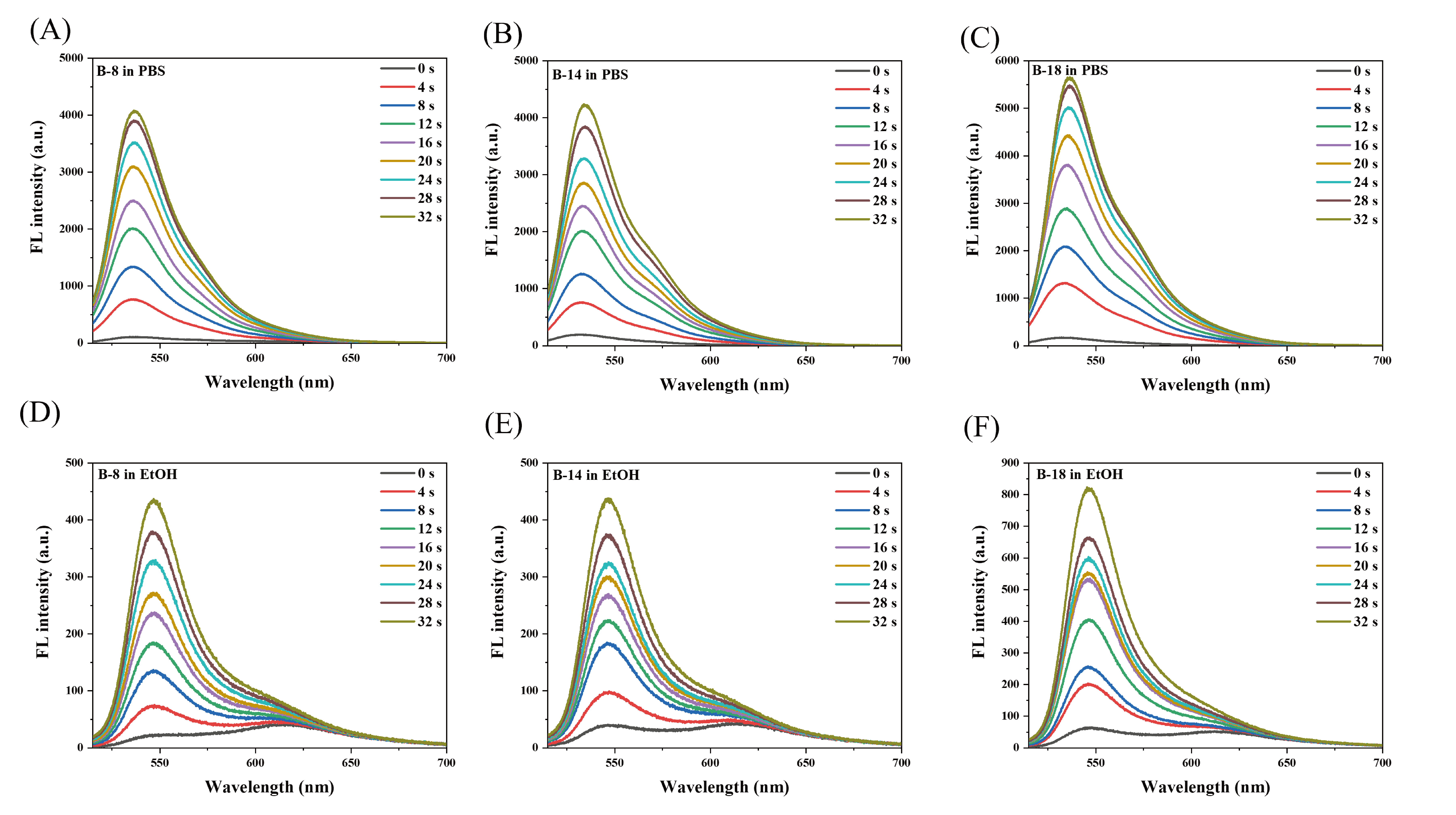


Figure S18. Fluorescent spectra of **B-8**(A)**,** **B-14**(B)**,** and (C)**B-18** with **DCFH** in PBS, and EtOH (D-F) at different light times (concentration: 10μM, light: 20 mW cm^−2^)

Table S1. Fluorescence quantum yields of BODIPY derivatives in different systems.

| Fluorescence quantum yield(ΦF) | EtOH (40%) | EtOH (1%) |
| --- | --- | --- |
| B-8 | 0.011 | 0.0074 |
| B-14 | 0.011 | 0.0001 |
| B-18 | 0.013 | 0.0000 |
